# A Fast Protocol for Multiparametric Characterisation of Diffusion in the Brain and Brain Tumours

**DOI:** 10.3389/fonc.2021.554205

**Published:** 2021-09-21

**Authors:** Ricardo Loução, Ana-Maria Oros-Peusquens, Karl-Josef Langen, Hugo Alexandre Ferreira, N. Jon Shah

**Affiliations:** ^1^Institute of Neurosciences and Medicine 4, INM-4, Forschungszentrum Jülich, Jülich, Germany; ^2^Institute of Neurosciences and Medicine 11, INM-11, JARA, Forschungszentrum Jülich, Jülich, Germany; ^3^Faculty of Medicine, RWTH Aachen University, Aachen, Germany; ^4^Institute of Biophysics and Biomedical Engineering, Faculty of Sciences of the University of Lisbon, Lisbon, Portugal; ^5^Jülich Aachen Research Alliance (JARA) – BRAIN – Translational Medicine, Aachen, Germany; ^6^Department of Neurology, RWTH Aachen University, Aachen, Germany

**Keywords:** diffusion MRI, trace, IVIM, kurtosis, fast acquisition

## Abstract

Multi-parametric tissue characterisation is demonstrated using a 4-minute protocol based on diffusion trace acquisitions. Three diffusion regimes are covered simultaneously: pseudo-perfusion, Gaussian, and non-Gaussian diffusion. The clinical utility of this method for fast multi-parametric mapping for brain tumours is explored. A cohort of 17 brain tumour patients was measured on a 3T hybrid MR-PET scanner with a standard clinical MRI protocol, to which the proposed multi-parametric diffusion protocol was subsequently added. For comparison purposes, standard perfusion and a full diffusion kurtosis protocol were acquired. Simultaneous amino-acid (^18^F-FET) PET enabled the identification of active tumour tissue. The metrics derived from the proposed protocol included perfusion fraction, pseudo-diffusivity, apparent diffusivity, and apparent kurtosis. These metrics were compared to the corresponding metrics from the dedicated acquisitions: cerebral blood volume and flow, mean diffusivity and mean kurtosis. Simulations were carried out to assess the influence of fitting methods and noise levels on the estimation of the parameters. The diffusion and kurtosis metrics obtained from the proposed protocol show strong to very strong correlations with those derived from the conventional protocol. However, a bias towards lower values was observed. The pseudo-perfusion parameters showed very weak to weak correlations compared to their perfusion counterparts. In conclusion, we introduce a clinically applicable protocol for measuring multiple parameters and demonstrate its relevance to pathological tissue characterisation.

## Introduction

The use of magnetic resonance imaging (MRI) is considered to be the standard clinical practice for non-invasive, *in vivo* brain tumour characterisation. Traditionally, T_1_-, before and after contrast agent administration, and T_2_-weighted images are acquired. Changes caused by contrast agents are based on changes in the T_1_ relaxation time, and fluid-attenuated inversion recovery (FLAIR) contrast is based on the lengthened T_2_ relaxation time in the tumour and oedema regions. However, these parameters are seldom measured directly and the presence of these changes is only assessed qualitatively.

In contrast, quantitative MRI (qMRI) enables the acquisition of parameters that do not depend on either the scanning protocol or the scanner, field strength notwithstanding. Applying such an approach to tumour lesion assessment results in more accurate evaluations and could, ultimately, improve diagnosis (1). Furthermore, qMRI facilitates a meta-analysis of results from different centres, enabling a greater breadth of research, i.e. larger cohort studies.

In brain tumours, tumour tissue generally becomes increasingly heterogeneous with disease progression (2). This heterogeneity is mainly seen at a microscopic level as different mutations in the cells result in regions of distinct underlying microstructure (3). Consequently, no single contrast is able to categorically characterise this whole range of differentiation, and, therefore, a multi-parametric approach to tumour segmentation and characterisation is required.

Several MRI measurable parameters can be used to probe aspects relevant to the changes in brain environment due to tumours [e.g. T_1_, T_2_, ${\text{T}}_{2}^{*}$, chemical exchange saturation transfer, magnetisation transfer, and diffusion MRI (dMRI) (4–7)]. Among these, dMRI is particularly useful as it is directly sensitive to different regimes of water mobility and thus to different microscopic environments of varying characteristic lengths that are well below the voxel dimension (8).

Typically, a dMRI experiment assumes that diffusion in tissue is Gaussian (9). This results in a mono-exponential signal decay which is given by:


(1) $$\frac{\text{S}(\text{b})}{\text{S}(0)}={\text{e}}^{-\text{b}\cdot {\text{D}}_{\text{app}}}$$

where b is the diffusion weighting value (b-value), S(b) and S(0) are the magnitude of the signal at diffusion weighting b and 0 s/mm^2^, respectively, and D_app_ is the apparent diffusivity (8).

When a diffusion sensitising gradient of low strength is applied (low b-values), the presence of a fast-decaying component in the diffusion-weighted signal is evident. This fast component is often interpreted as the water moving within randomly oriented capillaries. In the framework introduced in (10), this is known as intravoxel incoherent motion (IVIM). At diffusion weightings above b ≈ 200 s/mm^2^, this component is suppressed and a tissue-characteristic decay becomes apparent at higher b-values. The signal equation then becomes:


(2) $$\frac{\text{S}(\text{b})}{\text{S}(0)}=\text{f}\cdot {\text{e}}^{-\text{b}\cdot {\text{D}}^{*}}+(1-\text{f})\cdot {\text{e}}^{-\text{b}\cdot {\text{D}}_{\text{app}}}$$

where *f* is the perfusion fraction, D^*^ is the pseudo-diffusion coefficient, and D_app_ the apparent diffusivity. Previously, the perfusion fraction, *f*, has been considered to be related to cerebral blood volume (CBV), as obtained from dynamic susceptibility contrast (DSC) measurements, while the product *f.*D^*^ relates to cerebral blood flow (CBF) (11).

Since the contribution of D^*^ to the signal is relatively small [approximately 10% in the brain (12)], its effects can be neglected even at moderately low b-values (>200/mm^2^). The mono-exponential approximation of Eq.1, leading to an apparent diffusion coefficient ADC, is most often considered.

Due to the presence of microscopic barriers, which hinder the motion of water molecules, the apparent diffusivity of water in tissue is substantially reduced by a factor of three or more, compared to that of free water. This regime is still described by Gaussian diffusion and characterises the hindered motion of water in the extracellular space.

The application of Eq.1 is, however, limited by an upper b-value. For b-values above 1000 s/mm^2^, diffusion can no longer be considered Gaussian (13–15). This is due to the fact that the microstructure of tissue is highly heterogeneous and has many restrictive barriers to diffusion (16).

Diffusion in tissue can be characterised by a sum of two exponentials: one reflecting the slow tissue diffusion effects, and the other reflecting the fast tissue diffusion (13). At the voxel level, the convolution of these components leads to the observation of non-Gaussian diffusion (NG-diff). However, this behaviour is only clearly apparent at large b-values (> 3000 s/mm^2^) (13).

**Figure 1:**
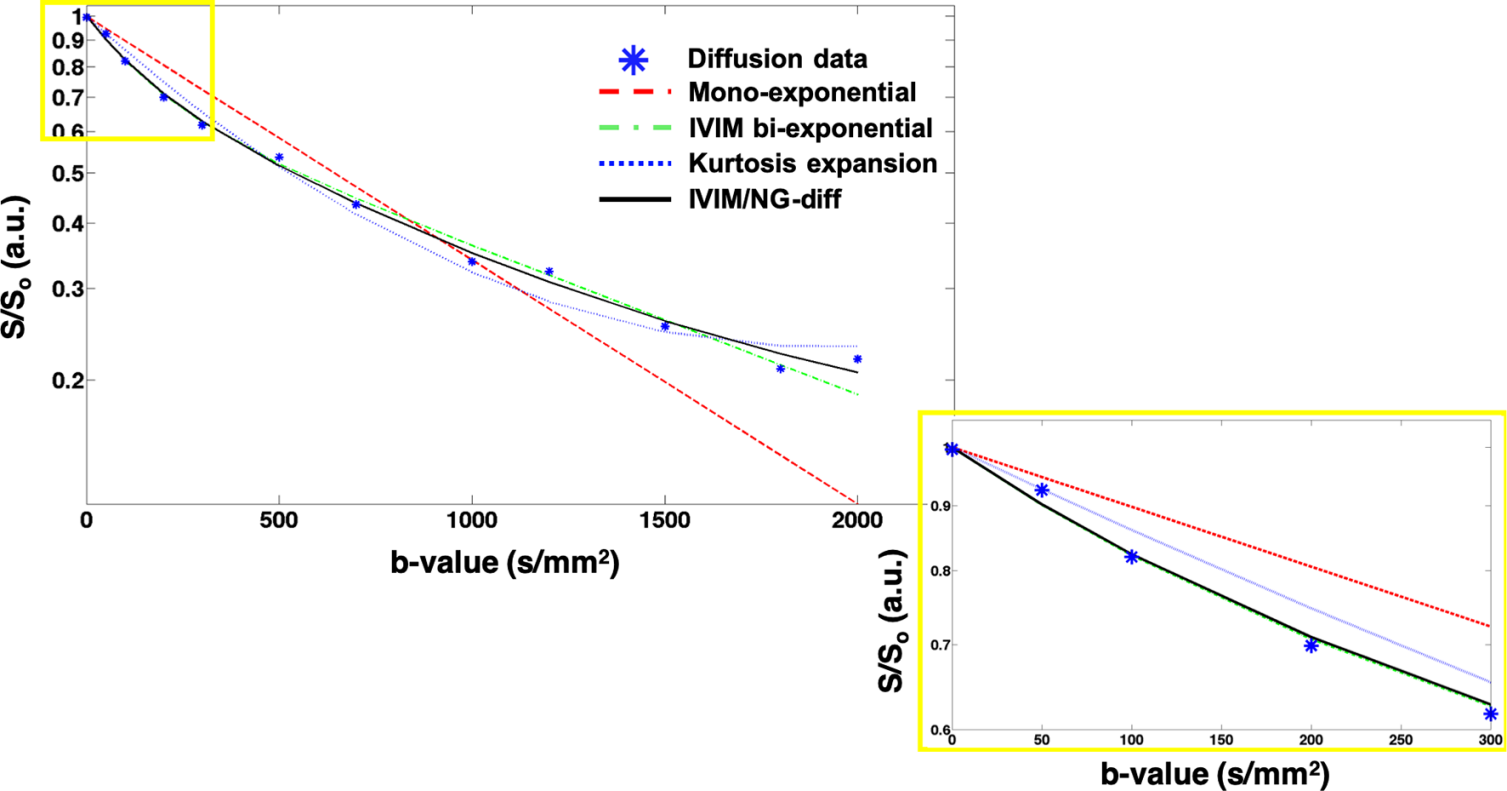
Signal decay *vs.* b-value between 0 and 2000 s/mm^2^. The lines represent a fitted curve taking into account the mono-exponential (red dashed), the IVIM bi-exponential (green dash dotted), the kurtosis expansion (blue dotted), and the combined IVIM/NG-diff (green line). The influence of IVIM is apparent in the lower b-value range (0-200 s/mm^2^) (inset), while at the higher b-values (1500-3000 s/mm^2^), the signal deviates from the mono-exponential, exhibiting evidence of kurtosis.

For an intermediate range of b-values, deviations from Gaussian-diffusion are often characterised by describing the first-order deviation from Eq.1 within the formalism of diffusion kurtosis imaging (DKI) (14), quantified by an additional term in the signal exponential:


(3) $$\text{S}(\text{b})=\text{S}(0)\cdot {\text{e}}^{-\text{b}\cdot {\text{D}}_{\text{app}}+\frac{1}{6}\cdot {\text{b}}^{2}\cdot {\text{D}}_{\text{app}}^{2}\cdot {\text{K}}_{\text{app}}}$$

where K_app_ is the apparent diffusional kurtosis coefficient. A plot of the signal in the different regimes is shown in **Figure 1**.

In the context of tumour assessment, both the intricacy of the microstructure and tissue irrigation are important parameters. Due to the rapid growth of tumour cells, angiogenesis is promoted in and around the lesion (17). This is typically assessed by measuring the effect of contrast-agent on the brain signal using high temporal resolution DSC. Increases in CBF, derived from DSC, have been observed in tumour regions (18, 19). Furthermore, previous studies have suggested that mean kurtosis (MK) can be successfully used to grade brain tumours due to its higher sensitivity to the tissue microstructure as compared to MD (20–22).

However, the images used to compute these parameters are often acquired separately and either require the administration of a contrast agent (as in DSC) or acquisition times that are too long to be practicable in standard clinical examinations (as with the use of a fully sampled DKI protocol). In order to address these limitations, we propose a fast hybrid IVIM/NG-diff protocol with the goal of being able to derive all of the aforementioned parameters in a clinically acceptable measurement time. By simultaneously acquiring this set of largely complementary parameters, a multiparametric approach to tumour characterisation can be achieved.

In this study, we propose a diffusion-weighted protocol based on the trace acquisition of 16 b-values, used to probe three diffusion regimes (IVIM, Gaussian, and non-Gaussian). Requiring roughly four minutes of acquisition time, the protocol is fast enough for standard clinical brain tumour imaging and is also fast enough for use in more time-stringent applications, such as sub-acute stroke.

We emphasise the fact that we use a diffusion acquisition based on the scanner’s ‘trace images’, which we will refer to in the following as a ‘trace-based design’. The ‘trace acquisition’ is based on the combination on the scanner of only three orthogonal diffusion weightings, and thus very seldom corresponds to the rigorously determined tensor-derived trace. It is, however, a diffusion measure commonly used in clinical practice and a reasonable first approximation of the proper trace in regions with low anisotropy.

We assessed the performance of the proposed method by comparing its results to those obtained from standard perfusion and non-Gaussian diffusion measurements. Preliminary results have been reported in (23–25).

Presently, no single MRI-derived parameter appears powerful enough to rival the specificity of positron emission tomography (PET) to identify active tumour tissue. An MRI-based quantitative, multiparametric approach to tumour characterisation might, however, achieve this goal. The present protocol is able to provide four parameters relevant to tumour environment (*f* and *f.*D^*^, as blood volume and flow surrogates, and apparent diffusivity and kurtosis, as microstructural probes) and can contribute to defining the unique quantitative multiparametric signature of each tumour. This could be particularly relevant for diagnosis, staging and treatment planning.

## Materials & Methods

### *In Vivo* Imaging

A cohort of 17 brain tumour patients was considered in this study (seven female, mean ± std age 46.2 ± 12.4 years old). Ethical approval was obtained from the University Hospitals of Aachen, Cologne and Düsseldorf in accordance with the requirements of the local ethics committees. Prior to scanning, written, informed consent was given by the patients. Patients underwent simultaneous PET and MRI measurements after referral to our centre from the above-mentioned hospitals. The measurements were acquired in a hybrid Siemens (Erlangen, Germany) scanner, based on a 3T Tim-TRIO MR system with a BrainPET insert (26).

The MRI dataset consisted of standard clinical protocols, such as high-resolution, volumetric T_1_-weighted pre (T_1_) and post gadolinium contrast (T_1_c), high-resolution volumetric T_2_-weighted (SPACE), and T_2_-weighted with fluid attenuation (FLAIR), and dynamic susceptibility contrast (DSC). Since the PET acquisition required patients to be in the scanner for 50 minutes, it was possible to include research protocols during the simultaneous MRI imaging, as well as a standard, clinically oriented examination. These research protocols included quantitative MRI scans, such as diffusion kurtosis imaging (DKI), multi-echo gradient echo (meGRE), further described in (27), as well as the proposed protocol (IVIM/NG-diff).

Relevant imaging parameters for the proposed protocol include: spin-echo echo planar imaging (SE-EPI) with TR/TE = 5100/92 ms, 3 orthogonal diffusion directions and 16 b-values (0, 50, 100, 200, 300, 500, 700, 1000, 1200, 1500, 1800, 2000, 2200, 2500, 2700 and 3000 s/mm^2^), with a voxel size of 2x2x2 mm^3^, 24 slices with a 1.4 mm slice gap, for a field-of-view (FOV) of 220x156 mm^2^, partial Fourier coverage of 5/8, iPAT of 2, and bandwidth of 909Hz/pixel, totalling an acquisition time of 4 mins 19 secs.

For comparison, a DKI protocol was adapted from one of clinical value used at our institute for measurements on brain tumour patients. To reduce distortions and echo time, the protocol was modified slightly to match the FOV and the orientation of the trace-base acquisition and the bandwidth was increased to the limit allowed by duty cycle constraints. Both protocols were based on standard Siemens sequences.

The DKI dataset was acquired with the following parameters: SE-EPI with TR/TE = 4000/115 ms, BW=1299Hz/pixel, no iPAT, 3 non-zero b-values (1000, 2000 and 3000 s/mm^2^), each with 30 non-colinear diffusion directions spread around the half-sphere. A FOV of 220x160 was used, with the same voxel size, number of slices, slice gap, and Fourier coverage as the proposed protocol. The acquisition time amounted to 6 mins 01 secs.

For perfusion assessment, a contrast-enhanced DSC T_2_*-weighted sequence was acquired. Single-shot EPI was used with TR/TE = 1500/32 ms, a voxel size of 1.79x1.79x5 mm^3^, 20 slices with a 1.75 mm slice gap and an image matrix of 128×128. The contrast agent (GdDTPA) was injected with a power injector (Injektron 82 MRT, Medtron AG), *via* an 18- to 20-gauge intravenous catheter at a dose of 0.1 mmol/kg of bodyweight (flow rate, 5 mL/s). Images were acquired continuously for 1 min.

Simultaneously with the MR protocols, amino acid O-(2-^18^F-fluoroethyl)-L-tyrosine (^18^F-FET) PET was acquired. The amino acid was produced *via* nucleophilic ^18^F fluorination with radiochemical purity above 98%, specific radioactivity greater than 200 GBq/mol, and a radiochemical yield of around 60% (28).

### Image Processing

**Figure 2:**
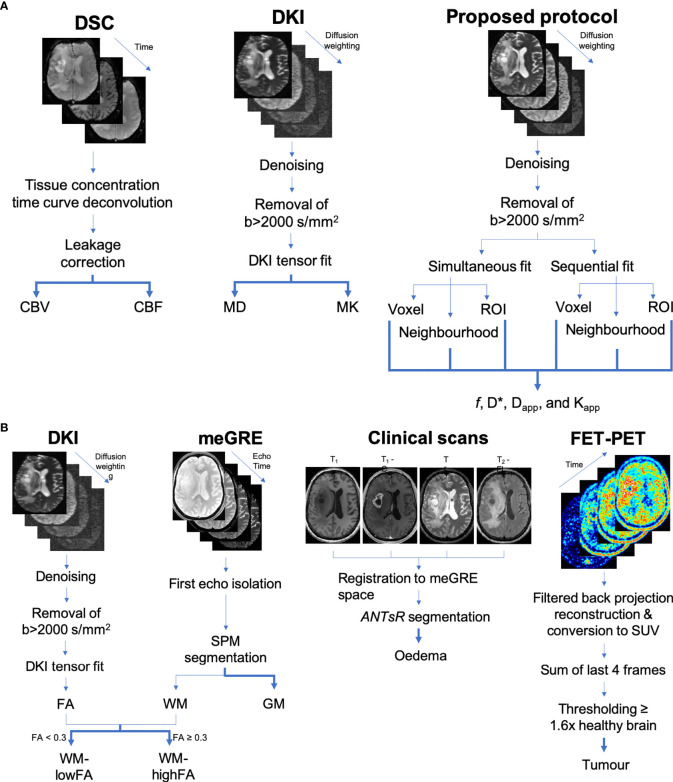
**(A)** Flowcharts of the processing steps leading to the computed maps. **(B)** Flowcharts of the processing steps leading to the computed tissue masks.

Flowcharts of the complete image processing pipelines can be found in **Figure 2A**.

To ensure similar data quality for all patients included in this study, the first step included a visual quality check on all data sets used.

Noise reduction was then performed on the multi-contrast diffusion data obtained from either the DKI or from the trace-based protocol, using a PCA-based algorithm (29). Its main features have been previously described for different types of multi-contrast acquisitions (30, 31) and are similar to those in the method proposed by (32), albeit with some differences – see **Appendix**.

For the data acquired with the DKI protocol, motion and eddy current artefacts were corrected using FSL’s *eddy* (33) and a Gaussian filter with full-width-half-maximum and a kernel size of 1.5 and 3x3x3 voxels, respectively, was applied as a final step.

The data from the proposed protocol were saved directly in trace form. Therefore, due to the lack of information on the directionality of the diffusion weighting, motion and eddy current compensation was performed using FSL’s *eddy_correct* (34). A Gaussian filter with the same properties as that used for the DKI data was subsequently applied. Finally, the noise floor was removed from the images by subtracting the average signal of the voxels in the image corners.

Use of parallel imaging iPAT=2 for the trace-based protocol helped with reducing the susceptibility-induced distortions in the acquired images, thus the performance of the two different algorithms (more powerful eddy and more basic eddy_correct) on the two different data sets led to very comparable results.

### Diffusion Data Fitting

The DKI data were used to estimate both the diffusion and kurtosis tensors and several rotationally invariant metrics, including mean diffusivity (MD), mean kurtosis (MK) and fractional anisotropy (FA), using United DKI (35).

In contrast, the IVIM/NG-diff data in the same b-value range were fit using three different approaches: a sequential fit, a simultaneous fit, and a region of interest fit.

The sequential fit obtains D_app_ from Eq. 2, using the assumption that the IVIM effect is negligible at b<200 s/mm^2^. Afterwards, *f* and D^*^ are sequentially determined, using the values previously obtained. Finally, K_app_ is estimated using a constrained variation of the Nelder-Mead simplex method (36), as implemented in MATLAB (R2014a, MathWorks, Massachussets, USA) *fminsearch* function, applied to Eq. 3. For greater detail, please see **Appendix**.

The second fitting approach (simultaneous fit) aimed to estimate all four parameters simultaneously (*f*, D^*^, D_app_, and K_app_) using the same constrained fitting routine based on the Nelder-Mead simplex algorithm. The target equation was then set to the following:


(4) $$\frac{S(b)}{S(0)}=f.e^{-b.D^{*}}+(1-f)e^{-b.D_{app}+\frac{1}{6}.b^{2}.D_{app}^{2}.K_{app}}$$

The constraints were used both in an attempt to mitigate the effects of local minima and to guarantee that each parameter belonged to a biologically plausible interval, based on existing literature. The perfusion fraction, *f*, was set to be between 0 and 0.3, D^*^ between 0.004 and 0.05 mm^2^/s (12), D_app_ between 0.0001 and 0.003 mm^2^/s, and K_app_ between 0 and 3 (37).

Both the sequential fit and simultaneous fit routines were conducted on a voxel-by-voxel (single voxel fit) basis and on a kernel basis (neighbourhood fit). The kernel used was an in-slice 3x3 neighbourhood around the central voxel, where the signal of the nine voxels was averaged and then fitted.

The third fitting approach used averaged signals from specific tissue classes (see *Tissue Classes* section). The averaged signal was fitted to Eqs. 1 (mono-exponential), 2 (kurtosis expansion), 3 (IVIM bi-exponential) and 4 (IVIM/NG-diff). This was done in order to investigate the necessity of including terms describing non-Gaussian diffusion in the fit when the signal-to-noise ratio (SNR) is high enough to unequivocally distinguish between models. The fitting parameters were obtained using the simultaneous approach, i.e. for each equation, all unknowns were determined using the same non-linear constrained fitting routine as in the simultaneous fit. Additionally, sequential fitting was also performed when fitting Eq. 4.

The fitting routines were performed on a MacBook Pro (early 2015), running Mac OSX 10.14.1 Mojave, with an Intel Core i5 2.7GHz processor and 16GB of RAM.

### Miscellaneous Processing

DSC data were processed using in-house built routines, as described in (38). The tissue concentration time curve was deconvoluted from the measured signal using singular value decomposition. The arterial input function was automatically derived based on time-to-peak and the signal fitting was corrected for leakage (39). Finally, maps of CBV and CBF were extracted.

PET data were reconstructed using a 3D filtered back-projection algorithm and later expressed as standard uptake value (SUV) (40).

Finally, all of the resulting maps were warped to the DKI space using affine transformations determined by SPM12 (41). Visual quality control was carried out at each step.

### Tissue Classes

The performance of this new protocol was assessed in the context of different underlying microstructures. For each subject, masks were generated for each of the five tissue classes considered as described below and as shown in the flowcharts of **Figure 2B**.

First, volumes from each modality acquired were manually divided into the hemisphere containing the tumour (ipsilateral) and the normal-appearing hemisphere (contralateral to the tumour). Information from the clinical protocols, DKI, and PET was included.

Normal-appearing grey (GM) and white (WM) matter probability maps were obtained using the meGRE images and SPM12 united segmentation (41). From these probability maps, a threshold of 98% was applied to generate the GM and WM masks. These masks were then warped to the DKI space, also using affine transformations.

The WM class was further divided into two. From the DKI data of the standard protocol, the FA maps were used to define two classes, low FA (0.05<FA<0.3), and high FA (FA>=0.3). These were then used to divide the WM mask into WM-lowFA and WM-highFA. Given the structure of white matter on a microscopic level, WM-lowFA includes voxels where fibre arrangements are complex, e.g. crossing or fanning fibres, mimicking isotropic diffusion at the voxel level, whereas WM-highFA refers to voxels where the fibres are very well aligned, resulting in highly anisotropic diffusion.

Active tumour tissue was identified using a high-SNR data set obtained from the sum of the last four frames of the dynamic ^18^F-FET scans. The tracer uptake in the WM of the hemisphere contralateral to the tumour was defined as normal tissue value. The voxels in the summed data set with an intensity equal or higher than 1.6x that of normal tissue were considered to be active tumour (40).

Oedema masks were obtained using the morphological data (T_1_, T_1_c, SPACE, and FLAIR) on the ANTsR framework (42). The algorithm relies on random forests to perform the segmentation and was trained using the data from the BRATS 2015 challenge, available from the Sicas Medical Image Repository (www.smir.ch) (43).

This process led to the creation of five masks (GM, WM-lowFA, WM-highFA, tumour and oedema), which, due to different resolutions, point spread functions and thresholding used for the different acquisition methods, might not be mutually exclusive. Mask overlap was then resolved in the following way: if a voxel belonged simultaneously to tumour and oedema masks, it was removed from the oedema mask; if a voxel belonged simultaneously to more than one mask of healthy appearing tissue, the voxel was also removed from the analysis.

### Simulations

Simulations were conducted to assess the influence of noise, tissue-specific parameters and fitting procedure in the estimation of the IVIM/NG-diff metrics. All simulations were implemented in MATLAB.

Firstly, using the same b-value array used in the *in vivo* acquisition and the results of the tissue class-based fit (see *Results* section **Table 1**), a theoretical signal was generated from Eq. 4. Then, five different levels of noise were added to the theoretical signal, such that the SNR ranged between 20 and 60 in increments of 10. Finally, each SNR level was fitted 10,000 different times, each iteration with independently drawn noise. The accuracy and precision of the results given by each fitting procedure were then assessed.

**Table 1:** Subject level averages for the tissue class fitting routines to the IVIM/NG-diff model (Eq. 13). Significant differences between the fits are found in *f* and D_app_ in all the classes, and in D^*^ in GM, (p-value < 0.05).

	Simultaneous Fit				
	GM	WM-lowFA	WM-highFA	Oedema	Tumour
***f***	0.13 ± 0.04	0.03 ± 0.02	0.03 ± 0.01	0.03 ± 0.02	0.01 ± 0.01
D^*^ **(x10^-3^ mm^2^/s)**	8.43 ± 2.65	21.62 ± 12.52	23.02 ± 13.84	28.98 ± 14.98	29.53 ± 17.79
**D_app_** **(x10^-3^ mm^2^/s)**	1.12 ± 0.12	0.94 ± 0.05	0.88 ± 0.04	1.42 ± 0.22	1.38 ± 0.41
**K_app_**	0.83 ± 0.04	1.03 ± 0.04	1.12 ± 0.05	0.74 ± 0.11	0.72 ± 0.22
	**Sequential Fit**				
	**GM**	**WM-lowFA**	**WM-highFA**	**Oedema**	**Tumour**
***f***	0.20 ± 0.04	0.10 ± 0.02	0.10 ± 0.01	0.14 ± 0.04	0.11 ± 0.04
D^*^ **(x10^-3^ mm^2^/s)**	12.16 ± 1.46	17.63 ± 3.89	17.69 ± 2.63	19.96 ± 9.30	25.92 ± 10.95
**D_app_** **(x10^-3^ mm^2^/s)**	0.86 ± 0.06	0.71 ± 0.03	0.66 ± 0.02	1.05 ± 0.15	1.06 ± 0.32
**K_app_**	0.83 ± 0.03	1.02 ± 0.04	1.11 ± 0.05	0.73 ± 0.10	0.73 ± 0.22

### Statistical Analyses

When comparing *in vivo* sequential and simultaneous fitting results, Spearman’s ρ correlation coefficients were obtained between all the IVIM/NG-diff metrics (*f*, *f*.D^*^, D_app_, and K_app_) and their canonical counterparts (CBV, CBF, MD, and MK, respectively).

In order to determine the value of the added terms to the mono-exponential representation in the description of the signal decay, corrected Akaike information criteria (AICc) (44) were obtained for the fits performed at the tissue class level. Lower AICc shows an improved relationship between the residuals of the fit and the information gain, i.e., models that represent the data better will have a comparatively smaller AICc.

A comparison between the means of the metrics derived from the sequential and simultaneous tissue class level signal fitting to Eq. 4 was performed using the Wilcoxon signed-rank test. This test was also performed to assess differences in the means of the simulation results between both fits at the different SNR levels.

The reproducibility of the fits was assessed by calculating the coefficient of variation (CV) for each parameter obtained from the simulations.

All statistical analysis was carried out in MATLAB. Hypothesis testing was conducted at a significance value of 95% (p-value < 0.05).

## Results

### Noise Reduction

**Figure 3:**
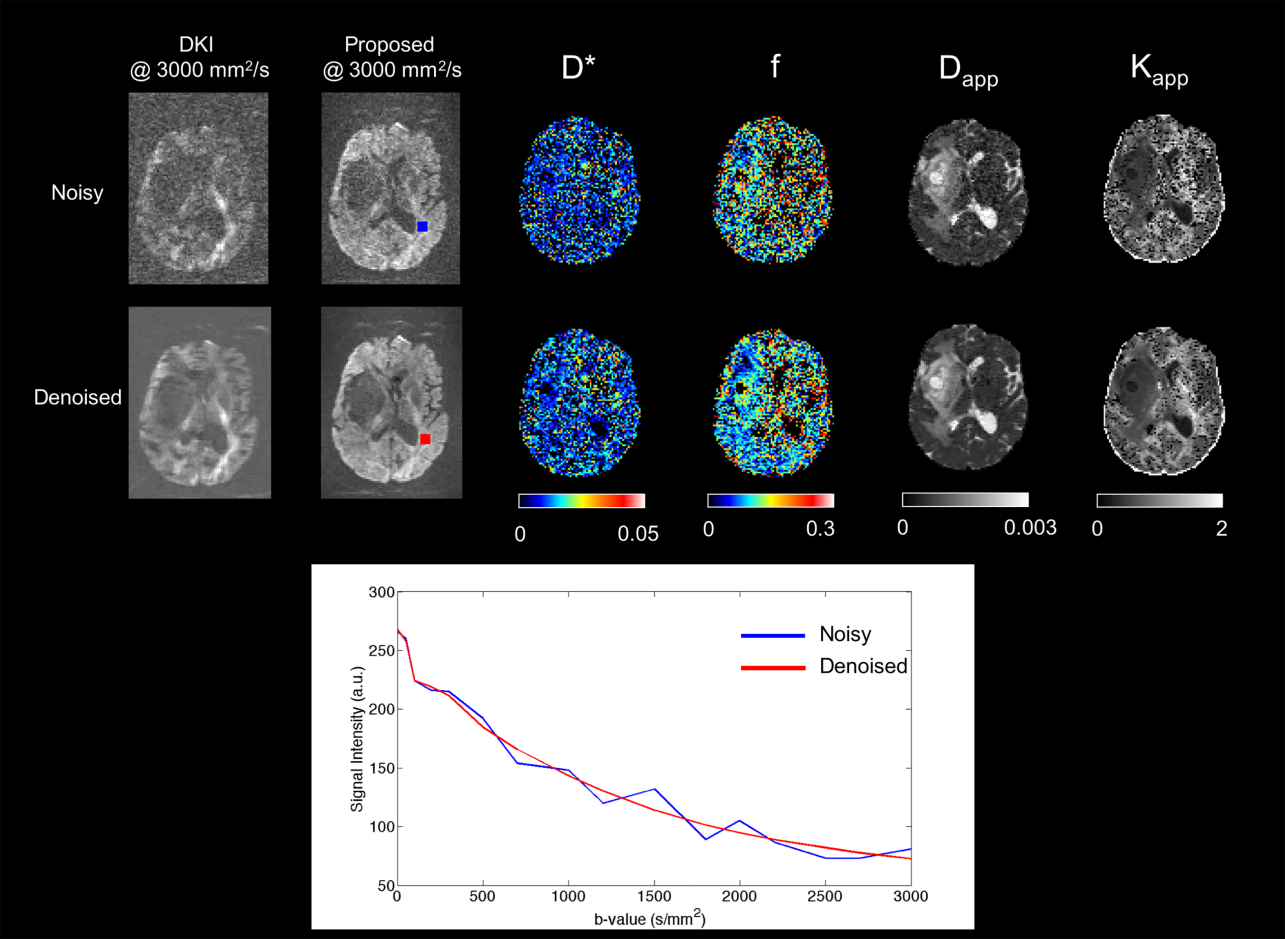
Effect of the denoising algorithm on the images and signal. The images of the top row are from the DKI acquisition while those of the bottom row are from the proposed protocol. All images are shown without the application of a gaussian filter. The plot under the images shows the signal decay of a white matter voxel before (blue) and after (red) denoising. The influences of the denoising are more apparent as the b-value increases.

In order to demonstrate the effects of denoising, **Figure 3** shows a representative slice taken from a brain tumour patient acquired with both DKI and IVIM/NG-diff protocols. From left to right, the images depict a slice at b = 3000 s/mm^2^, for both acquisitions; D^*^, *f*, D_app_, and K_app_ for the simultaneous fit. The signal decay of a WM-highFA voxel is also plotted against b-value, for the noisy (red) and denoised images (blue).

The singular value decomposition of the diffusion signal acquired in either protocol was found to be very stable across patients – see **Supplementary Figure 1**. Following confirmation by visual inspection, all components with a singular value below the value determined by this threshold were assigned to noise/artefacts and discarded.

### *In Vivo* Imaging

The mean ± standard deviation computation time of the fitting routines was 4.6 ± 0.8 ms for the sequential fitting and 27.7 ± 9 ms for the simultaneous fitting, per voxel.

**Figure 4:**
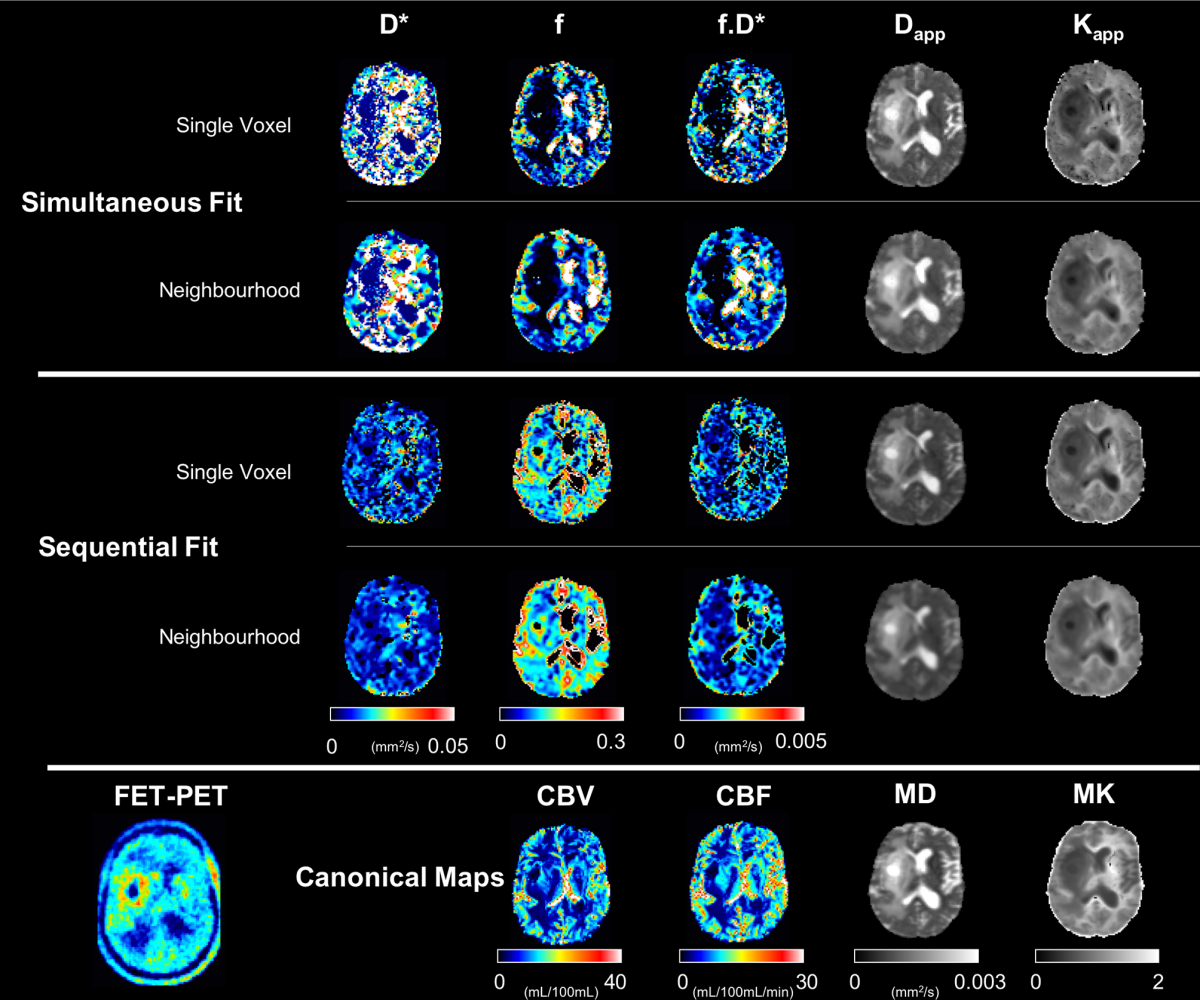
Computed maps with the proposed protocol and all fitting approaches for a representative subject. The canonical maps are shown in the last row. All scales within each metric are the same.

**Figure 4** shows maps of the five parameters estimated from the proposed protocol, as obtained by the simultaneous fit (upper block) and by the sequential fit (middle block), together with their canonical counterparts (lower block), on a representative patient and slice. The corresponding FET-PET slice is also shown in the bottom left corner. Additional patients are shown in **Supplementary Figures 2–7**.

The tissue class fitting results are summarised in **Table 2**, which shows the mean ± standard deviation metric values across all subjects for both fitting routines. The IVIM parameters are smaller when obtained from the simultaneous fit, as compared to those from the sequential fit, whereas the tissue D_app_ is larger. This is significant for *f* and D_app_ in all tissue classes, and for D^*^ in GM (Wilcoxon signed-rank test, p-value < 0.05).

**Table 2:** Mean ± standard deviations of all parameters for each fitting routine and tissue class across all subjects.

	*f*					*f.*D^*^ (x10^-4^ mm^2^/s)					D^*^ (x10^-3^ mm^2^/s)							D_app_ (x10^-3^ mm^2^/s)					K_app_				
	GM	WM-lowFA	WM-highFA	Oedema	Tumour	GM	WM-lowFA	WM-highFA	Oedema	Tumour	GM		WM-lowFA	WM-highFA		Oedema	Tumour	GM	WM-lowFA	WM-highFA	Oedema	Tumour	GM	WM-lowFA	WM-highFA	Oedema	Tumour
**Simultaneous Fit**	0.13 ± 0.02	0.06 ± 0.01	0.05 ± 0.01	0.03 ± 0.02	0.06 ± 0.03	21.40 ± 6.08	14.90 ± 5.24	13.91 ± 1.88	9.65 ± 5.29	13.80 ± 3.25	26.34 ± 6.35		37.51 ± 4.98	41.15 ± 4.96		36.28 ± 9.91	37.04 ± 8.34	0.90 ± 0.23	0.88 ± 0.05	0.85 ± 0.04	1.20 ± 0.26	1.20 ± 0.29	0.80 ± 0.03	1.00 ± 0.05	1.05 ± 0.05	0.68 ± 0.14	0.66 ± 0.12
**Sequential Fit**	0.14 ± 0.01	0.11 ± 0.01	0.10 ± 0.01	0.09 ± 0.02	0.12 ± 0.03	11.43 ± 0.96	9.11 ± 0.89	8.85 ± 0.80	6.82 ± 1.84	9.70 ± 1.70	8.37 ± 1.18		8.38 ± 0.73	8.57 ± 0.78		7.52 ± 1.33	8.65 ± 1.00	0.74 ± 0.19	0.69 ± 0.03	0.66 ± 0.03	0.97 ± 0.19	0.97 ± 0.21	0.79 ± 0.03	0.99 ± 0.04	1.06 ± 0.05	0.70 ± 0.13	0.69 ± 0.12
**Neighbourhood** **Simultaneous Fit**	0.14 ± 0.03	0.07 ± 0.02	0.05 ± 0.01	0.03 ± 0.02	0.06 ± 0.03	19.63 ± 5.95	12.48 ± 3.19	10.97 ± 2.05	7.98 ± 4.79	11.70 ± 3.04	20.78 ± 6.26		31.80 ± 6.47	37.24 ± 6.73		36.98 ± 11.44	36.73 ± 10.84	0.92 ± 0.24	0.91 ± 0.05	0.87 ± 0.04	1.26 ± 0.22	1.24 ± 0.22	0.82 ± 0.04	0.99 ± 0.04	1.06 ± 0.05	0.69 ± 0.13	0.70 ± 0.12
**Neighbourhood** **Sequential Fit**	0.15 ± 0.01	0.12 ± 0.01	0.10 ± 0.01	0.10 ± 0.02	0.13 ± 0.03	11.98 ± 0.97	8.73 ± 1.12	7.98 ± 0.92	6.73 ± 1.73	9.68 ± 1.78	8.08 ± 1.17		7.42 ± 0.83	7.48 ± 0.82		7.11 ± 1.34	8.00 ± 1.07	0.74 ± 0.19	0.70 ± 0.03	0.67 ± 0.03	1.00 ± 0.21	1.00 ± 0.21	0.81 ± 0.03	0.98 ± 0.04	1.05 ± 0.04	0.70 ± 0.13	0.70 ± 0.11
**Canonical** **Metrics**	18.49 ± 2.37	11.70 ± 2.30	9.88 ± 1.71	7.47 ± 4.64	12.70 ± 5.76	17.00 ± 8.34	10.34 ± 5.10	8.62 ± 4.23	9.21 ± 4.47	16.94 ± 4.11								1.13 ± 0.22	0.91 ± 0.06	0.85 ± 0.05	1.31 ± 0.30	1.32 ± 0.24	0.89 ± 0.06	1.21 ± 0.08	1.35 ± 0.06	0.79 ± 0.16	0.81 ± 0.18
	**GM**	**WM-lowFA**	**WM-highFA**	**Oedema**	**Tumour**	**GM**	**WM-lowFA**	**WM-highFA**	**Oedema**	**Tumour**								**GM**	**WM-lowFA**	**WM-highFA**	**Oedema**	**Tumour**	**GM**	**WM-lowFA**	**WM-highFA**	**Oedema**	**Tumour**
	**CBV (mL/100mL)**					**CBF (mL/100mL/min)**												**MD (x10^-3^ mm^2^/s)**					**MK**				
	**DSC**																	**DKI**									

The parameters from the proposed protocol are aligned with their respective counterparts from DSC and DKI.

**Table 2** summarises the mean ± standard deviation of the IVIM/NG-diff metrics per tissue class per fit across all subjects, as well as their canonical counterparts.

**Figure 5:**
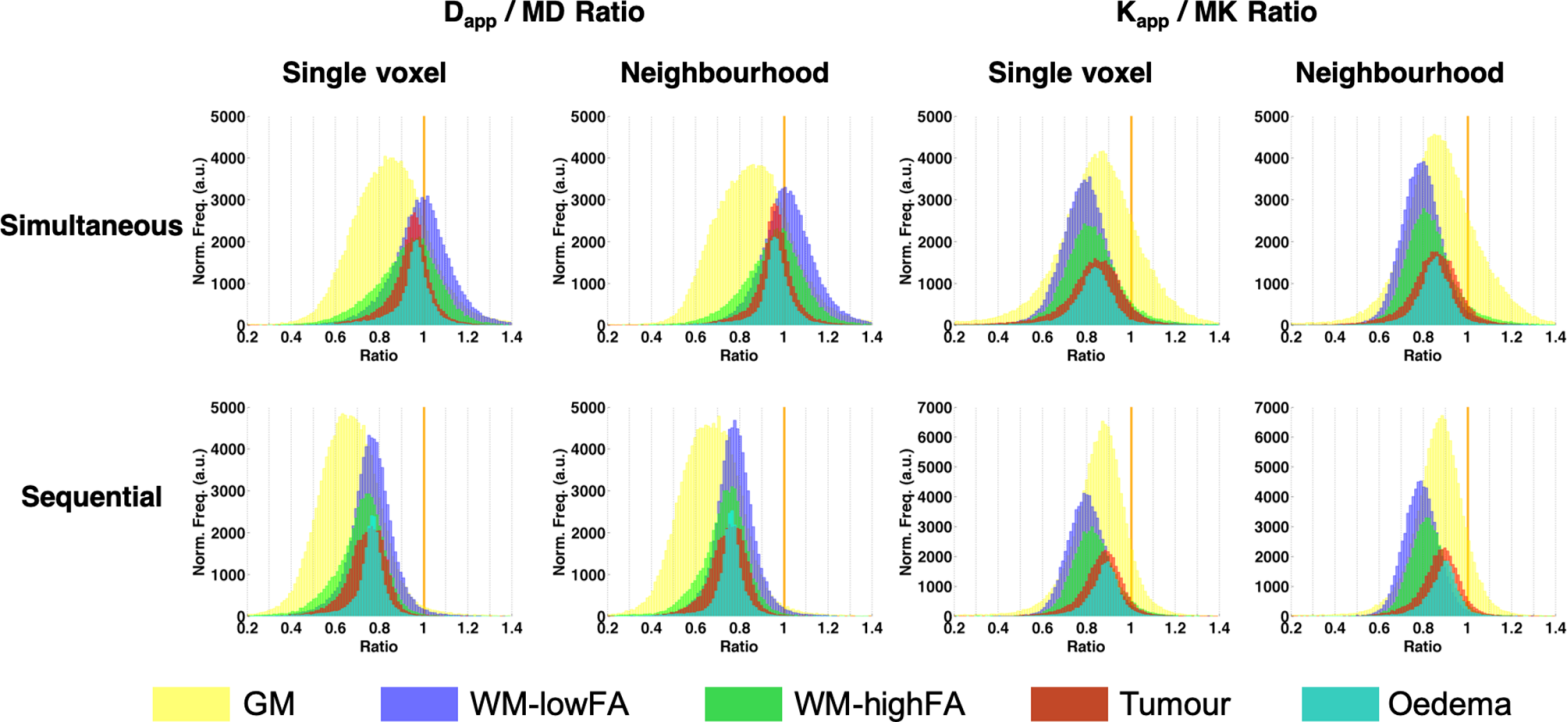
Ratio histograms of D_app_/MD and K_app_/MK. The vertical orange line indicates a ratio of 1.

**Figure 5** shows the voxel-by-voxel ratio histograms between the trace metrics and their respective tensor counterparts.

The Spearman ρ correlation values are summarised in **Table 3**. IVIM and DSC metrics showed weak correlations when the fits were performed using the simultaneous fit approaches (Spearman ρ ≈ 0.15). This is particularly evident when using the voxel-by-voxel fit, and even weaker correlations (Spearman ρ < 0.15) occur when using the sequential fit. The highest levels of correlations between IVIM and DSC are seen in the pathological tissues, with a Spearman’s ρ of around 0.2 in oedema when using the simultaneous fit.

**Table 3:** Spearman ρ correlation values.

	*f vs.* CBV				
	GM	WM-lowFA	WM-highFA	Oedema	Tumour
**Simultaneous Fit**	0.15 ± 0.06	0.13 ± 0.06	0.07 ± 0.05	0.21 ± 0.21	0.17 ± 0.09
**Sequential Fit**	0.15 ± 0.06	0.09 ± 0.06	0.08 ± 0.05	0.11 ± 0.31	0.15 ± 0.10
**Mean Simultaneous Fit**	0.12 ± 0.06	0.17 ± 0.08	0.11 ± 0.08	0.21 ± 0.18	0.18 ± 0.09
**Mean Sequential Fit**	0.13 ± 0.07	0.14 ± 0.08	0.12 ± 0.06	0.15 ± 0.30	0.18 ± 0.13
	*f*.D^*^vs. CBF				
	**GM**	**WM-lowFA**	**WM-highFA**	**Oedema**	**Tumour**
**Simultaneous Fit**	0.15 ± 0.05	0.10 ± 0.05	0.06 ± 0.03	0.12 ± 0.25	0.17 ± 0.12
**Sequential Fit**	0.13 ± 0.04	0.09 ± 0.02	0.09 ± 0.05	0.12 ± 0.13	0.15 ± 0.08
**Mean Simultaneous Fit**	0.16 ± 0.05	0.15 ± 0.06	0.10 ± 0.05	0.15 ± 0.26	0.22 ± 0.08
**Mean Sequential Fit**	0.15 ± 0.06	0.14 ± 0.04	0.10 ± 0.06	0.14 ± 0.25	0.20 ± 0.13
	**MD *vs.* D_app_**				
	**GM**	**WM-lowFA**	**WM-highFA**	**Oedema**	**Tumour**
**Simultaneous Fit**	0.64 ± 0.10	0.50 ± 0.09	0.46 ± 0.06	0.83 ± 0.07	0.80 ± 0.09
**Sequential Fit**	0.68 ± 0.09	0.54 ± 0.10	0.51 ± 0.08	0.84 ± 0.05	0.79 ± 0.09
**Mean Simultaneous Fit**	0.68 ± 0.09	0.56 ± 0.13	0.52 ± 0.08	0.86 ± 0.05	0.80 ± 0.09
**Mean Sequential Fit**	0.70 ± 0.08	0.61 ± 0.11	0.57 ± 0.08	0.86 ± 0.05	0.80 ± 0.09
	**MK *vs.* K_app_**				
	**GM**	**WM-lowFA**	**WM-highFA**	**Oedema**	**Tumour**
**Simultaneous Fit**	0.44 ± 0.12	0.53 ± 0.08	0.42 ± 0.11	0.68 ± 0.23	0.58 ± 0.15
**Sequential Fit**	0.70 ± 0.09	0.64 ± 0.10	0.49 ± 0.14	0.83 ± 0.12	0.68 ± 0.20
**Mean Simultaneous Fit**	0.52 ± 0.12	0.60 ± 0.09	0.49 ± 0.12	0.77 ± 0.19	0.61 ± 0.22
**Mean Sequential Fit**	0.76 ± 0.07	0.70 ± 0.10	0.55 ± 0.13	0.85 ± 0.16	0.73 ± 0.18

Diffusion metrics showed strong correlations overall with the DKI-derived parameters, with the lowest being WM-highFA using both sequential and simultaneous fit (sequential fit: diffusivity Spearman ρ=0.48 ± 0.10, kurtosis Spearman ρ=0.49 ± 0.11; simultaneous fit: diffusivity Spearman ρ=0.50 ± 0.10, kurtosis Spearman ρ=0.45 ± 0.11), and the highest being oedema (diffusivity Spearman ρ=0.89 ± 0.05; kurtosis Spearman ρ=0.84 ± 0.14).

**Figure 6:**
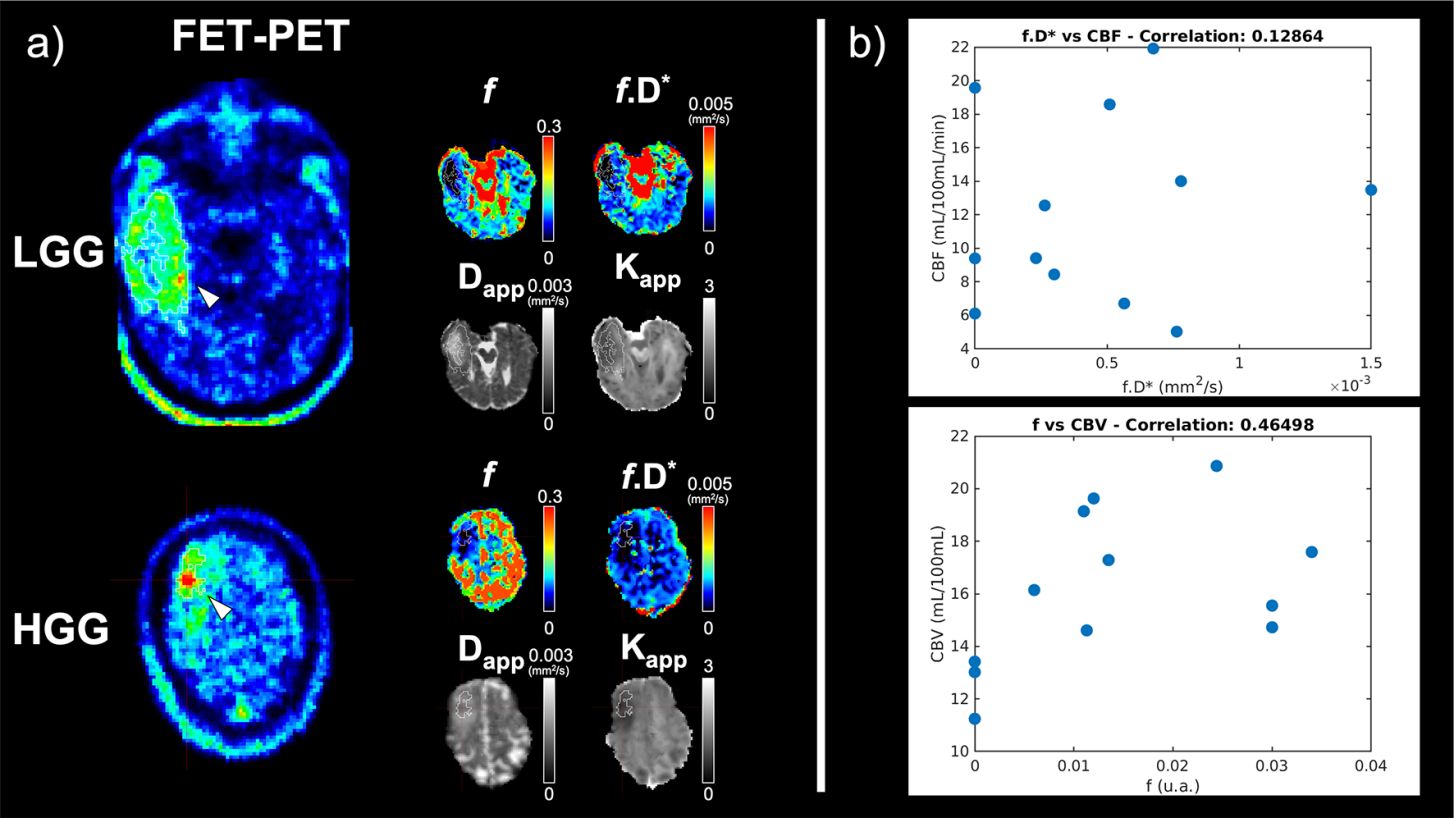
**(A)** Representative slices of a low-grade glioma (LGG) patient and a high-grade glioma (HGG) patient. FET-PET information is shown on the left and the corresponding slices of the maps from the proposed protocol following the mean neighbourhood fit are shown beside it. The tumour mask is outined in white. **(B)** Scatter plots of DSC-derived perfusion parameters cerebral blood volume (CBV) and cerebral blood flow (CBF), and the corresponding quantities derived from the present protocol (f and f.D*, respectively). An ROI-based fitting approach has been used. The size of the tumour ROI is included as a covariae when determining the strength of the correlation.

**Figure 6** shows data from two tumour patients (a low-grade and a high-grade glioma), together with scatter plots, correlating the perfusion metrics, with tumour size as co-variate.

### Simulations

**Figure 7:**
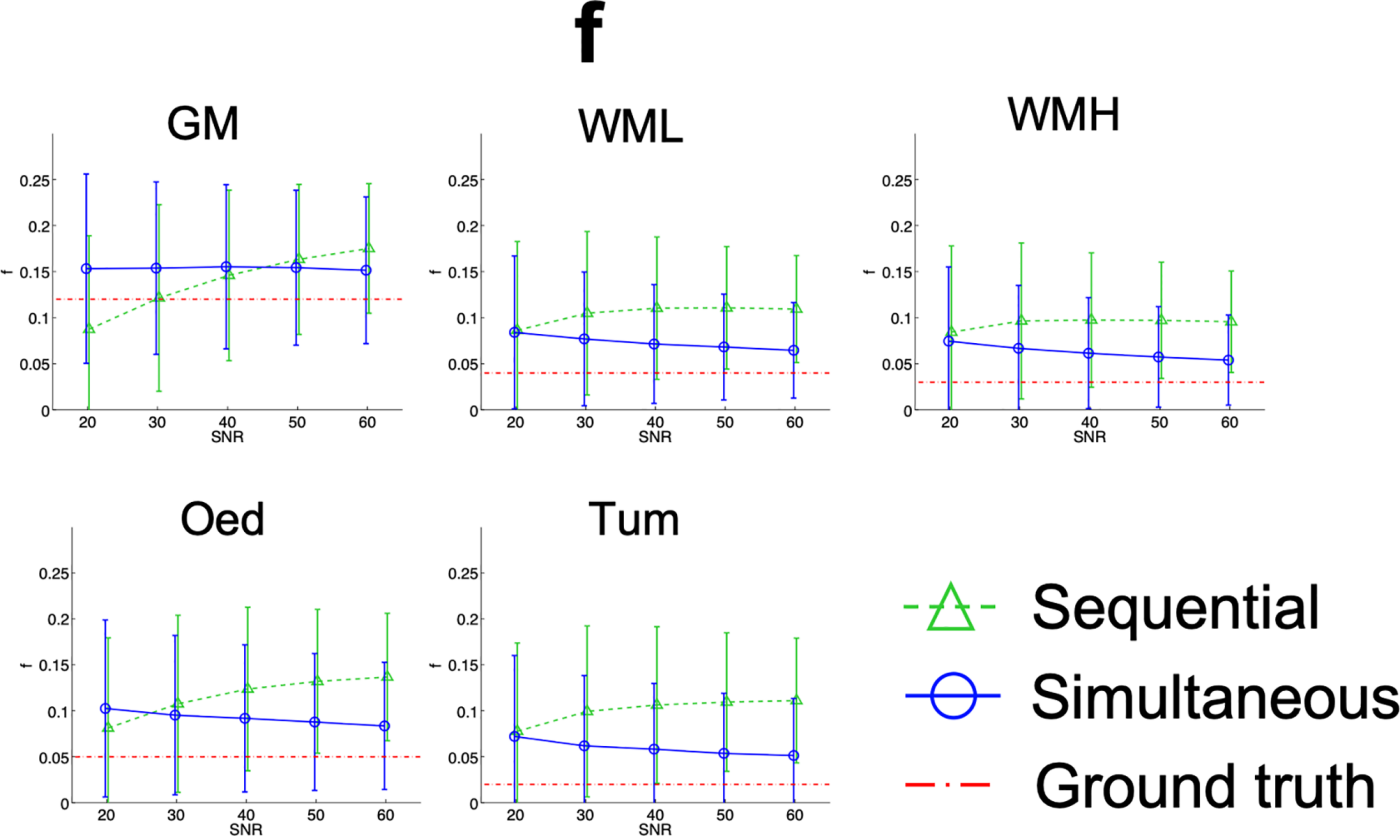
Simulation results plots of *f* in all tissues at all SNR levels. The geometric figures represent the mean and the vertical bars the standard deviation at each SNR level.

Results from the simulations are summarised in **Figures 7** **–** **10**, depicting the plots of the mean and standard deviation of the values obtained for each of the metrics, *f*, D^*^, D_app_, and K_app_ respectively, at each SNR level.

**Figure 8:**
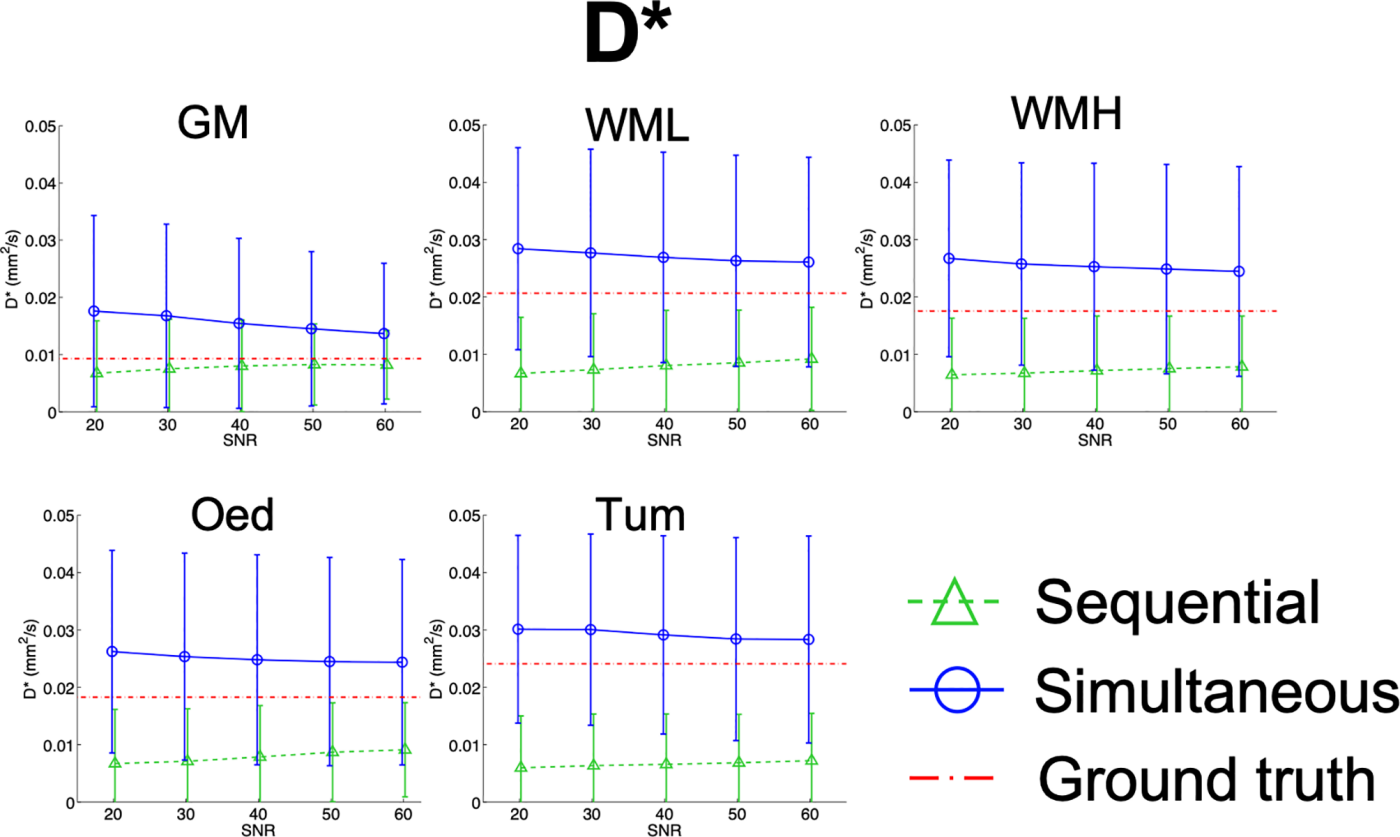
Simulation results plots of D^*^ in all tissues at all SNR levels. The geometric figures represent the mean and the vertical bars the standard deviation at each SNR level.

**Figure 9:**
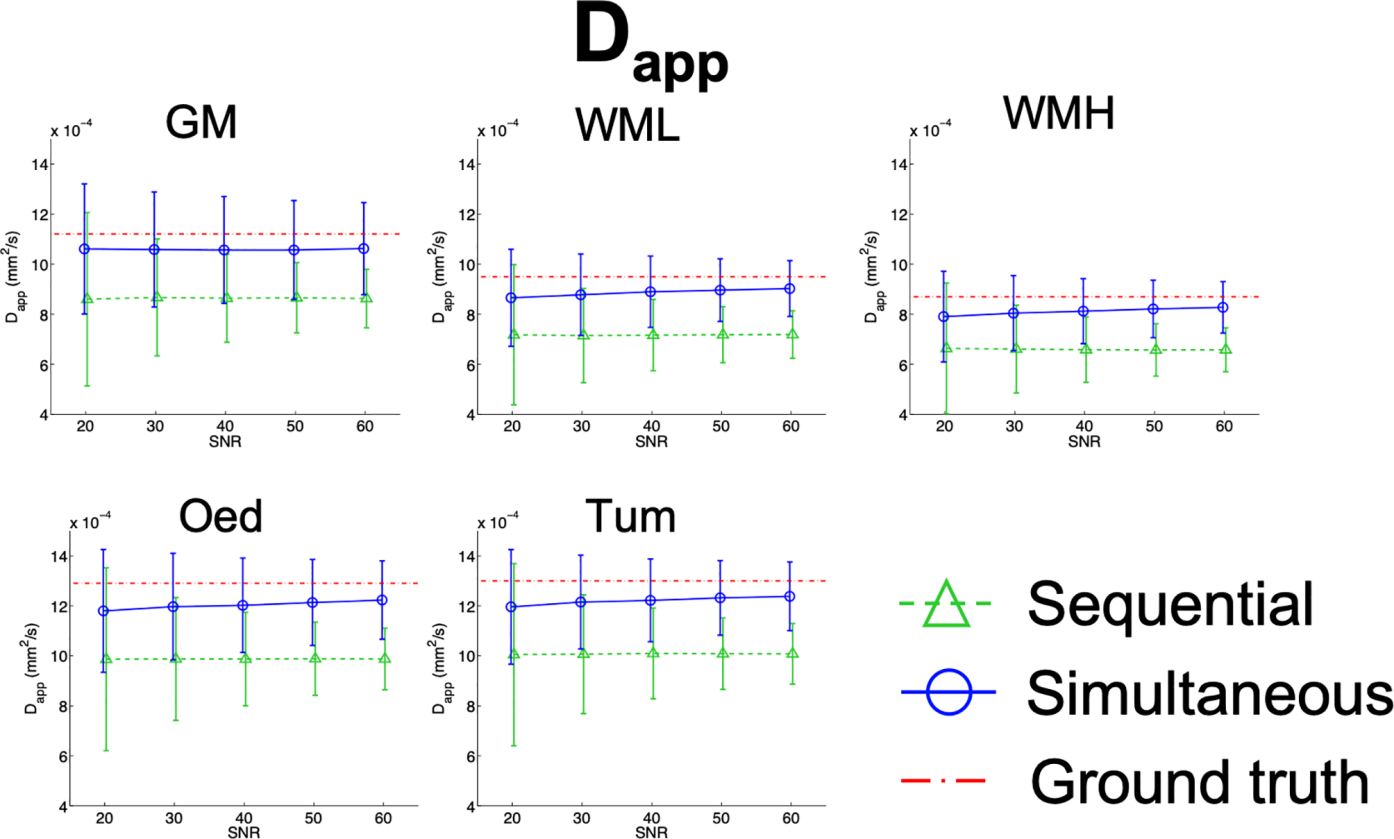
Simulation results plots of D_app_ in all tissues at all SNR levels. The geometric figures represent the mean and the vertical bars the standard deviation at each SNR level.

**Figure 10:**
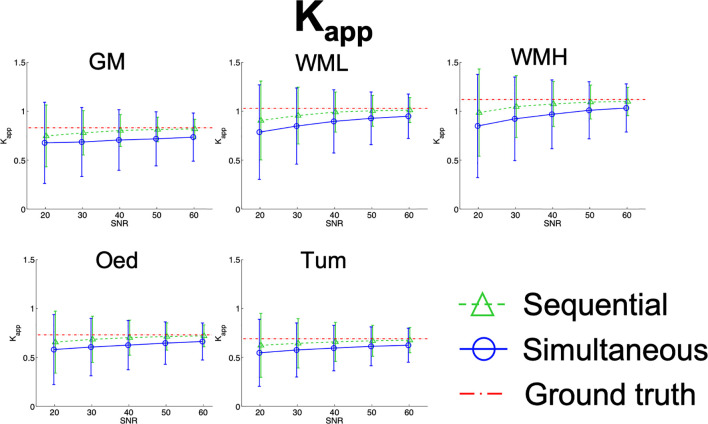
Simulation results plots of K_app_ in all tissues at all SNR levels. The geometric figures represent the mean and the vertical bars represent the standard deviation at each SNR level.

A broader range of considerations regarding the effects of sampling scheme, neighbourhood averaging and ROI-based averaging are included in **Supplementary Material: Additional Simulations**.

A more comprehensive display of the simulation results can be found in **Supplementary Table 1**. Here the means ± standard deviations of the distributions are shown, as well as the reproducibility of the metrics through means of CV and their relative error.

To summarise **Supplementary Table 1**, the precision of the parameter estimation increases with SNR (decrease in CV) for all metrics. In general, the simultaneous fit produces more precise results than sequential fitting, except for K_app_. Accuracy also increases with SNR and the highest accuracies were seen when estimating K_app_ using the sequential approach.

For comparison to the SNR range of the simulations, the mean ± standard deviation SNR of the acquired non-diffusion-weighted images was 51 ± 8 for the trace-based protocol and 60 ± 6 for the DKI protocol, ranging from 35 to 69.

For the Wilcoxon signed-rank results, the means of the distributions of the metrics were statistically different between the sequential and simultaneous fits across tissues and SNR levels, except for K_app_.

## Discussion

A clinically-aimed protocol for the simultaneous determination of IVIM and non-gaussian diffusion parameters is presented in this work. The protocol is based on the acquisition of trace-based diffusion data across a wide range of b-values in a manner intended to compensate for SNR loss at increasing b-values.

When using the “Trace” option for diffusion weighting with the manufacturer’s software, the geometric mean of three images acquired with orthogonal diffusion weighting directions is calculated on the scanner and output without allowing access to the original images. This is a commonly used contrast in clinical practice, usually with diffusion weighting of 1000 s/mm^2^, and our protocol has the advantage of including this widely used information. However, this is not rigorously speaking the trace of the diffusion tensor, except for fibres which are incidentally aligned with one of the orthogonal directions, or for voxels with isotropic diffusion on a macroscopic level. The latter is the picture generally used for IVIM (10). Measuring 6 directions instead of 3 and using a DTI formalism would allow us to derive a more reliable measure of the trace for voxels containing oriented fibres (high FA). However, this would double the measurement time and still be insufficient for a proper determination of the kurtosis tensor. Indeed, one can make the argument that 6 directions are insufficient for a proper characterization of even the DTI tensor (45). We have instead opted for sampling an extensive number of diffusion weightings while fully sacrificing the directional information.

The aim of the comparison kurtosis protocol was to determine how large the differences are between the ‘trace-based’ fit and a commonly used diffusion kurtosis protocol, which includes 30 directions and two non-zero b-values. The agreement between D_app_ and K_app_ derived in our model and MD and MK derived from the full DKI acquisition and tensor modelling is good (see for example **Figure 5**), especially so for tumour and oedema regions. We conclude that the ‘trace-based’ acquisition, even if imperfect regarding spherical invariance, appears sufficient to characterize the salient features of diffusion in brain tumours.

Since the ‘trace-based design’ has the advantage of including the clinically used ‘trace acquisition’ at b=1000 s/mm^2^, including it in the oncology routine would also be compatible with e.g. retrospective large-number patient evaluations based on common clinical protocols.

Other studies have started investigating faster diffusion routines (46, 47), and have shown their successful application to brain tumours (48). However, these acquisitions are tailored to determine the mean diffusivity and kurtosis alone. As there is growing interest in the multiparametric characterisation of pathological tissue in a variety of pathologies (49–54), the proposed protocol is presented in the context of an effort to acquire multiparametric, clinically relevant information in a short amount of time. Compared to the protocols in (46, 47), our proposed protocol has the advantage of enabling the analysis of IVIM metrics, at the cost of only a small increase in acquisition time.

One possible way to use these parameters simultaneously to inspect tissue properties is demonstrated in **Supplementary Figure 8**. The combination of the multiple parameters can be used to assess different pathological signatures, as shown in the radial plot in **Supplementary Figure 8**.

### Design of Acquisition and Denoising

It is well known that diffusion metrics like D_app_ and K_app_ are directionally dependent (46). However, FA in tumour tissue is greatly reduced (55), suggesting that the directional dependency decreases. This allows for the replacement of a shell-based acquisition with a faster, trace-based one, at least when the main goal is to characterise tumour properties.

Given the sensitivity of all the fit parameters to noise, and the fact that we aimed to describe three diffusion regimes by fitting their properties simultaneously, noise reduction in the diffusion data represents an important step in our approach to mapping tissue properties. One benefit of such an extensive multi-b-value protocol is that it makes it possible to exploit the redundancy of the acquired diffusion weightings to reduce noise in the data acquired with either the proposed or the standard DKI protocols. We address this redundancy briefly in the **Appendix**.

Denoising is achieved here by using PCA on the whole data set and then discarding components identified by several criteria as noise. This step has been demonstrated to improve the quality of the fit and its stability in the proposed method, as shown in **Figure 3**.

### Simulations

For low SNR levels, the averages of the IVIM metrics calculated from the simultaneous and the sequential fits differ to some degree, with the sequential fit being overall further from the ground truth. The exception to this is in the case of GM, as evidenced in **Figure 7**.

As SNR increases, a trend in the results of *f* emerges. Perfusion fraction calculated from sequential fit increases, moving away from the ground truth, while *f* from the simultaneous fit tends to converge to the ground truth.

In contrast, in **Figure 8**, D^*^ is shown to converge towards the ground truth with an increase in SNR, regardless of fit method.

Reproducibility of the IVIM metrics is generally low. This is exhibited by the high coefficients of variation and agrees with observations from the literature (56, 57).

The highest reproducibility is shown by D_app_ for both variants of the fit and is very similar at all SNR levels for the simultaneous fit (CV_Dapp@SNR20_SimFit_ = 24%, CV_Dapp@SNR60_SimFit_ = 17%). Bias was also seen in D_app_ when calculated with the sequential fit (**Figure 9**), which is confirmed by the *in-vivo* observations. Conversely, the simultaneous fit does not show this bias, as D_app_ approaches the ground truth with an increase in SNR.

With reference to K_app_, the two approaches are most divergent at lower SNR, where the simultaneous fit has a broader distribution than that of the sequential fit. This is evidenced in **Supplementary Table 1** where the coefficient of variation is shown (CV_Kapp@SNR20_SimFit_ = 62%, CV_Kapp@SNR20_SeqFit_ = 47%). Furthermore, the simultaneous fit shows a higher relative error than sequential fitting (Rel. Err_Kapp@SNR20_SimFit_ = 22%, Rel. Err_Kapp@SNR20_SeqFit_ = 11%), which is reduced with increased SNR. Finallly, both fits show a slight underestimation at lower SNR levels, which is then minimized at higher SNR, as shown in **Figure 10**.

It is worth noting that, for an SNR of 1000, the bias in the sequential fitting of *f*, D^*^, and D_app_ is still present (results not shown).

A potential cause for its presence is the sampling scheme, since the addition of extra low b-value information (b<100 s/mm^2^) would be beneficial for the proper determination of the IVIM parameters. However, in our experimental set-up, the scanner software does not allow for a finer sampling scheme in the sensitive interval, restricting the increment in b-values to 50 s/mm^2^.

To summarise, IVIM parameters, especially D^*^, are not very reliable even at high SNR. D_app_ is most reproducible but retains some bias. The parameter which is most accurately estimated is K_app_ but it shows lower reproducibility than D_app_ at lower SNR levels.

### *In Vivo* Acquisitions

The performance of the proposed protocol was evaluated by comparing the parameters derived here to their canonical counterparts. These metrics were extracted not only at a voxel level but also as a neighbourhood fit (averaging of the data in a small kernel) and whole tissue class level, in order to assess the validity of the IVIM/NG-diff model at a sufficiently high SNR *in vivo*.

The protocol proposed here requires a shorter acquisition time (4min:19s) than the combined acquisitions for DKI (6min:01s) and DSC (1min) information. This is, however, not considered to be the main advantage. Indeed, shorter DKI protocols have been proposed (52, 53) and could be used. Rather, the proposed protocol provides a more complete characterisation of grading-relevant tumour properties in a short measurement time, and could be easily extended to cover higher b-values and characterise the slow diffusion component of tissue. Instead, the kurtosis formalism is only applicable up to moderate b-values of 2500s/mm^2^.

The computation times of the two fitting approaches were very different, with the simultaneous fit taking nearly seven times longer than the sequential fit. Fitting the signal in a sequential manner has the advantage of speed, which is an important factor for making the fit results directly available on the scanner. When individual points are used in fitting, the computations are not very time expensive. The disadvantage of the approach is that by using very few points, the noise contribution on these few signals has a larger influence on the results. Simultaneous fitting, on the other hand, is more stable to noise contributions, to some extent, by virtue of the algorithm used, but this is at the cost of increased computational time.

All of the maps derived from voxel-based fits of the signal were very noisy with regard to the IVIM parameters (see **Figure 5**). The neighbourhood-based fit reduced the spatial variability of the parameters, but the IVIM-based maps still show little anatomical consistency.

At the tissue class level, the mono-exponential fit shows relatively high RMSE and AICc (**Supplementray Table 2**). By including the additional exponential term of the IVIM, the RMSE drops substantially and exhibits a lower AICc. Including both the IVIM and the kurtosis expansion further reduces both the RMSE and AICc. This shows that the combined IVIM/NG-diff is the best performing model amongst those considered.

These two results combined suggest that, despite the application of a denoising algorithm and Gaussian smoothing, the SNR at the voxel level might still be too low for such a complicated fit model. When averaged over the whole neighbourhood and especially at the tissue class level, SNR is increased sufficiently to reveal the necessity of including the IVIM term in the signal description.

Regarding the IVIM *in vivo* values, the healthy tissue averages obtained from the sequential fitting results are higher than those found in the literature, while tumour tissue average was within literature range (12, 58, 59) (*f*_WM_ = 0.03-0.09, *f*_Tum_ = 0.08-0.15). However, IVIM values obtained using the simultaneous fit procedure in healthy tissue are within the range of the literature but are lower than reported literature values in tumour tissue.

The correlation between IVIM and DSC metrics was poor, as seen in **Table 3**. This result does not support the hypothesis that IVIM can act as a surrogate for DSC. In fact, literature showing correlations between IVIM and DSC metrics in the brain is non-conclusive (60). Many studies report good correlations between IVIM and DSC, but some show poor or even negative correlations (60). This can be a result of many confounding factors. One of these factors is that IVIM and DSC can be sensitive to different phenomena, and therefore provide different information (60).

Whereas the high diffusivity of the additional IVIM term strongly suggests a vascular and flow-related origin, how well this additional term corresponds to perfusion is an open question, which we tried to address by comparison to DSC data.

Despite the fact that the IVIM-derived maps of blood volume and flow obtained with voxel-based fit methods show little similarity to the DSC-based counterparts, the correlation increases substantially when we compare values obtained from an ROI-based fit. The plots are shown in **Figure 6B**, and the correlation coefficients are 0.46 for CBV *vs. f.*D and 0.13 for CBF *vs. f.*D*, changing to 0.40 for CBV *vs. f* and 0.24 for CBF *vs. f.*D* when the tumour size is taken as a covariate. This shows that the influence of SNR on the precision/accuracy of the fit is still noticeable even when averaging over several hundred voxels. We note that IVIM-derived measures of blood flow, which involve D*, are less reliable than IVIM-derived measures of blood volume, reflected by *f*, due to the instability of the fit. The fact that the correlation between IVIM-derived blood volume and its DSC counterpart is modest (R=0.4) even when performing an ROI-based fit suggests that these parameters may indeed be different, and using both of them together might help discriminate between tumour types.

The two contrasts might reflect different aspects of the vasculature in different ways. IVIM generally refers to blood microcirculation, but other sources of intravoxel incoherent motion are possible (10, 11). For example, incoherent intravoxel dephasing can also appear in larger vessels, considering that laminar flow (or even more turbulent flow) leads to a distribution of velocities within the vessel lumen and to an IVIM signal attenuation which could be much larger than the perfusion-driven IVIM effect.

Furthermore, DSC results are also not equally sensitive to all vessel sizes, but emphasize large vessels [(38, 39) and refs within], such that the discrepancy between the two methods could also be due to an emphasis of the microvasculature in brain tumours. Also, the quantitation of DSC is influenced by extravasation of contrast agent. In this case the CBV is underestimated if T_1_-weighted effects induced by increased permeability of tumor vessels dominate, or overestimated if T_2_*-weighted effects dominate [(38, 39) and refs within].

Nevertheless, the estimated IVIM parameters support the notion that capillary density is higher in GM than in WM (61), where higher *f* values are seen in comparison to the rest of the brain. Regardless of the fit method, the average IVIM quantities have similar trends to those of their DSC counterparts across all tissue classes (highest in GM, lowest in oedema).

In oedema, the excess water in the tissue is probably mainly in the extracellular space (62), contributing to increasing D_app_. The lower *f* measured here (**Table 1**) might reflect this redistribution of water across the different compartments. In tumour tissue, vascularisation and perfusion are highly heterogeneous (17). IVIM parameters are therefore expected to differ considerably depending on tumour type, stage, and region, which could allow such parameters to be used in tumour grading. In fact, IVIM parameters have already been shown to be relevant in the evaluation of brain tumours (63–65) and breast lesions (66). This notion is somewhat supported here by the relatively higher standard deviation in both *f* and *f*.D* in the tumour tissue class. A grading analysis would be very enlightening in this respect but was precluded here by the small number of patients available for this study and the heterogeneity of the cohort.

D_app_ and K_app_ were found to have systematically lower values than their counterparts from the kurtosis tensor (see **Figure 5**), while still showing strong correlations with the DKI-derived parameters.

This is likely due to the fact that the IVIM fraction present in S(0) is not accounted for in either the diffusion tensor imaging (DTI) or the DKI models, whereas we have explicitly corrected for it in the IVIM/NG-diff model (e.g. Eq. 13). As a result, a stronger signal decay is described by the DTI/DKI models, leading to higher MD and/or MK values. Furthermore, differences in MD and FA obtained from conventional diffusion tensor imaging (DTI) and DKI have been reported (67). When the metrics were derived from the Gaussian DTI *vs* the non-Gaussian DKI tensors, differences of around 8% and 23%, and 1% and 17% were found in MD and FA, respectively (67). In this study, MD is derived using information from the signal decay up to a b-value of 2000 s/mm^2^, by means of the DKI tensor. This range of b-values is also used by the simultaneous fit of the proposed protocol. Conversely, D_app_ based on the sequential fit only uses information up to b=1000 s/mm^2^, much like a DTI fit would. The differences between the kurtosis and diffusion fits are also present in this study. In **Figure 5**, the histograms of the D_app_/MD ratio show an increased bias for all tissues when the fit is performed with the sequential method (similar to DTI) relative to that seen when the fit is performed with the simultaneous method (similar to DKI).

Another important factor is that the TE used in each protocol (TE_IVIM/NG-diff_ = 92 ms *vs* TE_DKI_ = 115 ms) was also slightly different even though the echo times used were the shortest allowed by the scanner. The diffusion signal decay is dependent not only on b-value but also on TE, and longer TEs have been shown to lead to overestimation of MD (68). The effect of TE is also an important consideration in the IVIM acquisitions. A study done on the prostate (69) has shown that both *f* and D* significantly increase with an increase in TE. This is due to the fact that the bi-exponential model of IVIM does not account for the different T2 values from blood and tissue. This can have important implications for *in vivo* brain applications.

A dense sampling scheme with around 20 b-values in the IVIM-relevant interval was used for simulations in order to mimic an appropriate experimental setup. Results of the simulations support the intuitive picture that a dense sampling scheme at a clinically achievable SNR is equivalent to sampling less points but at a higher SNR, obtained for example by averaging. The latter can be done either by repeating the acquisition, thus increasing the measurement time, or by averaging the signal over voxels with similar parameters. A number of 8 averages produces very similar results as the simulated dense sampling scheme in terms of fit accuracy and precision (**Supplementary Figures 5, 8 of the Supplementary Material: Additional Simulations**). Thus, the neighbourhood-based fit, which uses the signal averaged over 9 voxels, is expected to approximately compensate for the sparsity of the b-value sampling. This, however, holds only for regions which are homogeneous over the 3x3 voxel neighbourhood, such as WM. Even after increasing SNR by neighbourhood averaging, starting from our experimental initial SNR value of around 50, the simulations show that the precision and accuracy of the voxel-based fit of IVIM parameters is modest. The coefficient of variation for f is around 25% after neighbourhood averaging at an initial SNR of 50, that for D* is around 50%, while the systematic deviations are at around 15%. The situation becomes, however, increasingly better when SNR is increased by averaging over several hundred or even thousand voxels, which is the case for the ROI-based approach. Indeed, the tumours included in this study had active tissue volumes ranging from 108 to 10,611 voxels, as determined from FET-PET. For SNR values obtained by averaging over a homogeneous region of 1000 voxels with the perfusion properties of GM, the coefficient of variation for f is at 10%, with negligible systematic deviation, while D* still retains a coefficient of variation of 30% and bias of 8-10%. The fit becomes fully reliable in the IVIM regime for averages over 10,000 voxels or equivalently an SNR of 1500-2000. We reiterate that these very large SNR values are required for fit reliability of the IVIM parameters due to the small number of b-values sampled in the relevant interval in our protocol, and also due to the fact that the IVIM fraction is small in brain tissue, at the level of 10% or less. For an organ with a substantially higher perfusion fraction, for example 30%, the fit precision/accuracy would improve by roughly a factor 3 with the same sampling scheme and SNR, as shown by the simulations.

### Limitations

The bias observed in the IVIM quantities obtained by simulations points towards a shortcoming in the fitting and/or sampling procedures. The determination of D_app_ using the slope of the logarithm of the signal in the Gaussian diffusivity range is impacted by which b-values are included (70). In this study, we used a slope between b_1_ = 500 s/mm^2^ and b_2_ = 1000 s/mm^2^, which was considered to offer the largest dynamic range for signal attenuation due to diffusion in tissue and the lowest influence from IVIM effects.

The number of b-values acquired in the IVIM regime is smaller than those often used in the literature (12, 58, 59). Since the protocol is meant to be used in a clinical context with minimal changes to pre-existing sequences, the b-values chosen were limited by the manufacturer’s defaults: minimum b-value of 0 s/mm^2^, minimum increment of 50 s/mm^2^. However, this leads to too few data points with which to perform a proper fit of the IVIM signal. This limitation is especially true when trying to determine D^*^.

Partial-volume effects were not considered in our assessment of tissue class diffusion parameters. A voxel size of 2x2x2 mm^3^, as used here, can lead to some voxels containing more than one tissue class and can bias the estimated diffusion parameters for each class (71). Inclusion of mixed tissue classes in the simulations can help identify the degree to which the proposed protocol and processing routines are affected by this effect.

Since the data of the proposed protocol are saved in trace form, state-of-the-art routines like *eddy* could not be run. Instead, eddy current correction had to be performed with *eddy_correct*, which has been demonstrated to be outperformed by *eddy* (72). If the directional data from the proposed protocol had been saved individually, *eddy* could be used.

Finally, despite the considerations that lead to a trace-based design, NG-diff metrics derived from the proposed protocol remain non-rotationally invariant. Due to the small number of diffusion-encoding directions, a proper sampling of the micro-architecture is not possible, which could lead to a further bias in the results (45).

## Conclusions And Outlook

Here we present a protocol for joint IVIM/NG-diff acquisition. This pilot study aimed to assess the feasibility of adding several diffusion parameters for the characterisation of tumours within a short measurement time. We investigated IVIM, apparent diffusivity, and non-Gaussian diffusion characterised by apparent kurtosis. The *in vivo* validation of these parameters was performed by contrasting them to similar quantities derived from established protocols.

Non-gaussian diffusion metrics obtained from the proposed protocol were highly correlated with those obtained using standard DKI-derived metrics. Contrary to our initial premise, IVIM metrics were poorly correlated with DSC metrics, suggesting that they partly reflect different aspects of tissue. It was also shown that, to some extent, the parameters obtained from our protocol are reflective of tissue physiology.

Whereas each of the diffusion regimes (IVIM, Gaussian diffusion, and kurtosis) were assessed with respect to their grading qualities (18–22) and were found to be useful, to a greater or lesser extent, the multiparametric approach has not yet been fully exploited. We expect that a better characterisation of tumours will become possible by combining information from multiple diffusion regimes. Furthermore, b-values above 2000 s/mm^2^, which are outside the range of applicability of kurtosis model and were not fully exploited in this report, could be used to gain deeper insight into tumour microstructure.

In summary, we proposed a protocol which is stripped down to the minimum in terms of directionality sampling, but covers the relevant b-value range well enough to allow for a simultaneous IVIM-diffusion-kurtosis fit, is fast enough to be included in clinical evaluations, contains established clinical information (trace at b=1000s/mm^2^) and provides characterisation of tumour/oedema tissue (D_app_ and K_app_) which is similar to that obtained with a commonly used kurtosis acquisition (MD, MK). This implies that the tumour grading power of both parameters is kept to a large extent when using our protocol, and in addition also the IVIM parameters, shown to have grading power of their own (63–66), can be determined with reasonable precision and accuracy when using an ROI-based fit. Even for the latter case, we have shown here that the correspondence between IVIM and DSC-based perfusion characterisation is not very high (R=0.4 for blood volume and 0.23 for blood flow), suggesting that use of both parameters will give a more complete description of tumour tissue. A way of representing this multiparametric information to visualise a ‘tumour signature’ is suggested in **Supplmentary Figure 8**, but validating its significance for tumour grading would require a much larger data set, including PET and/or histological tumour characterisation. Of course, more quantitative parameters could be added to the description, such as R_2_* and water content, as proposed by our group (27).

The discussion of the proposed protocol was built around its usefulness for a deeper characterisation of brain tumours, which are notoriously heterogeneous and difficult to grade using MRI alone. It is anticipated that such a protocol will enhance the multiparametric assessment of tumour lesions. However, diffusion properties are certainly relevant to other pathologies and also to healthy tissue. A multi-b-value protocol such as ours, adapted to cover the whole brain at high resolution when the measurement time constraints are not as stringent as in clinical applications, will certainly prove a useful tool for understanding brain microstructure *in vivo*.

## Data Availability Statement

The raw data supporting the conclusions of this article will be made available by the authors, upon reasonable request.

## Ethics Statement

The studies involving human participants were reviewed and approved by ethical committees of RWTH University Hospital Aachen, Aachen, Germany; University Hospital Düsseldorf, Düsseldorf, Germany; and University Hospital Cologne, Cologne, Germany. The patients/participants provided their written informed consent to participate in this study.

## Author Contributions

RL contributed with the development of the method, data processing, and manuscript writing. A-MO-P contributed with development of the method, data acquisition, and manuscript writing. K-JL contributed with additional data acquisition. HF contributed with interpretation of data. A-MO-P, K-JL, HAF, and NS contributed with revision of the manuscript. All authors contributed to the article and approved the submitted version.

## Conflict of Interest

The authors declare that the research was conducted in the absence of any commercial or financial relationships that could be construed as a potential conflict of interest.

## Publisher’s Note

All claims expressed in this article are solely those of the authors and do not necessarily represent those of their affiliated organizations, or those of the publisher, the editors and the reviewers. Any product that may be evaluated in this article, or claim that may be made by its manufacturer, is not guaranteed or endorsed by the publisher.
