# Supplementary material for: A Fast Protocol for Multiparametric Characterisation of Diffusion in the Brain and Brain Tumours
Source: Front Oncol. 2021 Sep 21;11:554205. doi: 10.3389/fonc.2021.554205 (PMC8490752; doi:10.3389/fonc.2021.554205)
Supplement: Supplementary file 1 [file DataSheet_1.docx]

**Appendix**

Denoising details

Denoising was performed using a PCA-based algorithm. Specific to each type of data, a global cut-off was chosen for the number of components describing the signal (see the Results section) and the same cut-off was also used in a multi-scale approach.

By setting the small singular values to zero, matrix approximations of the data can be obtained, where the rank equals the number of remaining singular values (see below).

Schematically, the approach is as follows: the (n_x_,n_y_,n_z_,n_c_) 4-dimensional array is converted to a 2-dimensional M=(n_x_*n_y_*n_z_, nc) matrix. Here n_x_, n_y_, n_z_, n_c_ are the data dimensions in x, y, z and contrast, respectively. The singular value decomposition of this matrix is

$$M=USV^{*} (A.1)$$

Here the columns of U are the left-singular vectors of M, those of V are right-singular vectors and S is a diagonal matrix with elements s*_i_*, the singular values (eigenvalues). Each term u*_i_*s*_i_*v*_i_*^T^ is called a principal image or component.

The fraction s*_i_*^2^/sum(s*_i_*^2^) characterises the amount of variability in the data described by the *i*-th component. M is then approximated by a rank r matrix obtained from the first r components. The cut-off value can be estimated in a number of ways using, for example, visual, threshold-based, or random matrix theory-based methods [1, 2].

For the principal component analysis of DKI data, the contrast dimension amounted to 91 (all b-values and directions). Based on visual inspection, as well as on defining a threshold for the gradient of the eigenvalues, the number of signal-containing components was set to 16. The remaining components, assigned to noise and artefacts, were discarded by setting the corresponding eigenvalues to 0.

For the IVIM/NG-diff protocol, the number of contrasts n_c_ was 16. The number of signal-containing components, r, was set to eight, using the same gradient threshold as for the DKI data. The number of voxels (n_x_, n_y_, n_z_) was the same for both imaging approaches.

Data fitting details – sequential fit

The D_app_ was initially calculated by considering

$$\frac{S\left( b \right)}{S\left( 0 \right)}=f\cdot e^{-b{\cdot D}^{*}}+\left( 1-f \right)\cdot e^{-b\cdot D_{app}} \left( A.2 \right)$$

At b-values above 200 s/mm^2^, the contribution of the IVIM term is negligible so the signal equation becomes

$$S\left( b \right)=S\left( 0 \right).\left( 1-f \right).e^{-b.D_{app}} (A.3)$$

From this, the logarithm of the signal is taken, resulting in

$$log\left( S\left( b \right) \right)= -b.D_{app}+const (A.4)$$

where

$$const=log\left( S\left( 0 \right).\left( 1-f \right) \right) (A.5)$$

Equation A.4 is a straight line with slope D_app_. By taking two points from the slope within the valid b-value range (200-1000 s/mm^2^), D_app_ can be calculated by

$$D_{app}=-\frac{log\left( S\left( b_{2} \right) \right)-log\left( S\left( b_{1} \right) \right)}{b_{2}-b_{1}} (A.6)$$

where *b_1_* and *b_2_* were set at 500 and 1000 s/mm^2^, respectively. Once D_app_ is determined, *f* can be determined by substituting D_app_ in Eq. A.3, leading to

$$f=1-\frac{S\left( b_{2} \right)}{S\left( 0 \right)}.e^{b_{2}.D_{app}} (A.7)$$

D^*^ can then be calculated by revisiting Eq. A.2 and considering the wider b-value range of (0-1000 s/mm^2^). Rearranging Eq. A.7 in two different ways results in

$$S\left( 0 \right).f=S(0)-S(b_{2}).e^{b_{2}.D_{app}} (A.8)$$

and

$$1-f=\frac{S\left( b_{2} \right)}{S\left( 0 \right)}.e^{b_{2}.D_{app}} \left( A.9 \right)$$

By replacing Eqs. A.8 and A.9 in Eq. A.2, pseudo diffusion D^*^ can be derived by

$$D^{*}=\frac{log\left( S\left( 0 \right)-S\left( b_{2} \right).e^{b_{2}.D_{app}} \right)-log\left( S\left( b_{3} \right)- S\left( b_{2} \right).e^{{(b}_{2}-b_{3}).D_{app}} \right)}{b_{3}} (A.10)$$

where *b_3_* was set to 50 s/mm^2^. This value is selected among those sampled in the relevant interval as being the one where the IVIM effect is most prominent.

In order to fit K_app_, points in the signal with a b-value under 800 s/mm^2^ were removed and the remaining subset of points was non-linearly fit to Eq. 3 using a constrained variation of the Nelder-Mead simplex algorithm [3], as implemented by MATLAB’s *fminsearch* (R2014a, MathWorks, Massachusetts, USA), in which D_app_ was also allowed to vary.

Simulations details

All five different tissue classes were considered, each with a distinct signal decay, derived from a distinct set of parameters (*f*, D^*^, D_app_, and K_app_). These parameters were chosen as the results of the tissue class-based fit of eq. 4 of the manuscript (see Results – Table Ia).

The observed signal was calculated including noise contribution:

$$S_{N}= \sqrt{{{(S}_{T}+\eta_{Re})}^{2}+\eta_{Im}^{2}} \left( A.11 \right)$$

where S_N_ is the observed (noisy) signal, S_T_ the underlying tissue signal and η_Re_ and η_Im_ the real and imaginary noise components, respectively. These noise components were determined based on a Gaussian distribution with zero mean and a standard deviation of 1/SNR. The SNR range considered was 20 to 60, in increments of 10. This takes into account the SNR levels typically obtained in our data. At each SNR level, 10,000 independent noise samples were drawn, and the resulting data were fitted in the two ways described in the processing section, i.e. sequential fit and simultaneous fit. The accuracy and precision of the results given by each fitting procedure was then assessed.

Statistical analysis details

The AICc was determined by:

$$AICc=nlog\left( \hat{o}^{2} \right)+2k+ \frac{2k\left( k+1 \right)}{n-k-1} (5)$$

where n is the number of points used in the fit, k is the degrees of freedom in the model and ô^2^ is mean squared error, given by

$$\hat{o}^{2}= \frac{1}{n}\sum_{i=1}^{n} \varepsilon_{i}^{2} (6)$$

in which ε_i_ are the residuals of the fit at each data point.

The CV is given by:

$$CV= \frac{\sigma}{\mu} (7)$$

where σ and μ are the standard deviation and the mean, respectively, of the distributions for each metric.

Data redundancy and denoising considerations

Assuming that spherical invariance is ensured by the acquisition, or that diffusion weighting is performed in one orientation only, the b-value dependence of the signal can be described by a sum of three exponentials, corresponding to IVIM [4], fast, and slow tissue diffusion [5], leading to a total of six independent parameters. In the absence of noise, only six data points covering the relevant interval would be required to determine the diffusion properties. These are S0, *f*, D^*^, f_slow_, f_fast_, D_fast_, and D_slow_, where the sum of the fractions *f*, f_slow_, and f_fast_ is 1_._

When the directionality aspect is also considered, the number of parameters becomes higher, rising to at least eight in the case of collinear fast and slow-diffusion tensors. In this case, three eigenvalues of the diffusion tensor (assumed to be the same for fast and slow diffusion, apart from a scaling factor) replace the scalar value. The acquisition, however, becomes much more extensive as it is necessary to ensure the spherical invariance of the derived parameters [6]. If one goes beyond the diffusion tensor model, 15 parameters are required to determine the kurtosis tensor, in addition to the 3 describing IVIM.

Diffusion data acquired on clinical scanners have long TE and are noisy, especially at high b-values. Consequently, substantially more than six points must be acquired in order to enable proper parameter fitting.

The denoising performance is based on the redundancy of the acquired contrasts, which is decreased when the directionality aspect is included. This is due to the complicated fibre structure of white matter.

The effect of decreased denoising performance is reflected in the principal component analysis of the data. The number of components required to describe a single-shell acquisition of the DKI data is only marginally lower (by two components) than that required for the three-shell acquisition performed in our study (results not shown).

The denoising effect, defined as $\sqrt{\frac{n_{c}}{r}}$ (n_c_ = number of contrasts, r = rank of approximation), increases with the number of acquired contrasts in a similar way as averaging, once n_c_ >> r. The denoising performance is, thus, greater for the DKI data, with 91 contrasts acquired. If the same number of contrasts, n_c_ = 16, were acquired in the DKI as in the trace-based protocol, the denoising performance of PCA would be minimal for the DKI data, whereas a $\sqrt{2}$ value can already be obtained for the trace-based data.

Thus, it appears beneficial from the point-of-view of acquisition, denoising properties, and simplicity of fitting, to reduce the acquisition to a trace-based one with approximate spherical invariance and to sample an extensive range and number of b-values. Here, we have chosen numbers of b-values for each diffusion interval that approximately compensate for the decreasing SNR with b-value. The results suggest that denoising performance should also be included as a variable in the protocol optimisation for future applications.

Our measured protocol includes b-values up to 3000 s/mm^2^, which were sampled for two purposes. One was to assess the increase in deviations from spherical invariance of the trace with increasing b-value. The other was to investigate the feasibility of bi-exponential fitting of the tissue diffusion properties, thus going beyond the kurtosis model. Due to measurement time constraints, it was not possible to include an appropriate acquisition with 20-30 directions per b-value and several b-values, which would provide data for bi-exponential fitting and spherical invariance of the parameters. Such an acquisition would be required in order to serve as a standard for comparison with the trace-based model. Consequently, in this report, we have concentrated on the more frequently used kurtosis model. A standard DKI acquisition with 30 directions per b-value was used as a comparison with which to assess the performance of the trace-based protocol. The b-value interval relevant for the comparison covers up to a b-value of 2000 s/mm^2^, for which the kurtosis model provides a good approximation of the signal decay [7].

References

1. Jolliffe, I.T. (2002) “Principal Component Analysis (Springer Series in Statistics)”, Springer, ISBN13: 9780387954424
2. Veraart, J., Fieremans, E., Novikov, D. S. (2016) “Diffusion MRI noise mapping using random matrix theory”, Magn. Reson. Med., vol. 76, pp. 1582-1593, doi:10.1002/mrm.26059
3. Jeffrey C. Lagarias, James A. Reeds, Margaret H. Wright, Paul E. Wright, "Convergence Properties of the Nelder-Mead Simplex Method in Low Dimensions", SIAM Journal of Optimization, 9(1): p.112-147, 1998.
4. Le Bihan, D., Breton, E. (1985) “Imagerie de diffusion in-vivo par résonance magnétique nucléaire”, Comptes-Rendus de l'Académie des Sciences, vol. 93(5), pp. 27-34
5. Maier, S. E., Mulkern, R. V. (2008) “Biexponential analysis of diffusion-related signal decay in normal human cortical and deep gray matter”, Magnetic Resonance Imaging, vol. 26(7), pp. 879-904, doi: 10.1016/j.mri.2008.01.042.
6. Jones, D.K. (2004) “The effect of gradient sampling schemes on measures derived from diffusion tensor MRI: a Monte Carlo study”, MRM, vol. 51(4), pp. 807-815, doi: 10.1002/mrm.20033
7. Lu, H., Jensen, J. H., Ramani, A., Helpern, J. A. (2006) “Three-dimensional characterization of non-gaussian water diffusion in humans using diffusion kurtosis imaging”, NMR in Biomedicine, vol. 19(2), pp. 236-247, doi: 10.1002/nbm.1020
