# Supplementary material for: A Fast Protocol for Multiparametric Characterisation of Diffusion in the Brain and Brain Tumours
Source: Front Oncol. 2021 Sep 21;11:554205. doi: 10.3389/fonc.2021.554205 (PMC8490752; doi:10.3389/fonc.2021.554205)
Supplement: Supplementary file 2 [file DataSheet_2.docx]

**IVIM / Non-Gaussian diffusion simulations**

The simulations were conducted to assess the influence of SNR, b-value sampling strategy, perfusion amount, averaging, and size of ROI.

*Effect of SNR*

The simulations were conducted at two different SNR ranges. Firstly, an SNR range typically seen in the clinical environment of 20 to 60 (clinical SNR range), in intervals of 10. For context, the SNR levels in our study ranged from 35 to 69, with a mean of 51. Then, a second SNR range from 200 to 1000, in intervals of 200 (high SNR range).

Figures 1 and 2 show the percent error vs SNR for the clinical and high SNR ranges, respectively, while in figures 3 and 4 are plotted the coefficient of variation vs SNR for the clinical and high SNR ranges, respectively.

*Effect of b-value sampling*

Two different b-value sampling strategies were also simulated: the proposed strategy, described in the main article (proposed scheme); and a dense sampling scheme ranging from 0 to 2000 s/mm^2^ in intervals of 10 s/mm^2^ (dense scheme).

The relative percentual errors vs SNR for the dense scheme can be found in figure 5, and the coefficient of variation in figure 6.

*Effect of perfusion amount*

To test the perfusion amount/tissue type, simulations were conducted using different ‘ground-truth’ curves, for the different tissues considered. The tissues included those in the main text (GM, WM with high FA, WM with low FA, oedema, and tumour) and an additional fictitious tissue with high perfusion fraction (f=0.3) and all other parameters were kept the same as those in GM.

The simulation results for the different *in vivo* tissues can be seen in the main body of the article, while in figure 7 the relative percentual error is shown for the GM and high perfusion tissue.

*Effect of averaging and size of ROI*

The simulations were conducted without averages (i.e. one decay per fit), and with 8 averages (i.e. 8 signals independently sampled, averaged and then fitted). The results of averaging are shown in figure 8.

Since averaging increases the effective SNR of the data, and the goal of this work is to help provide a tissue characterizing parameter (which can be obtained by averaging the signals of neighbouring voxels), we additionally investigated the relationship between the tumour size, effective SNR, and accuracy of parameter estimation. To this end, simulations were conducted using 40 sets of voxels (ROI) with volumes ranging from 50 to 200,000 voxels, sampled from a logarithmic scale. For context, the tumour volumes seen in this study ranged from 108 to 10,611 voxels. Each ROI was fitted 10,000 times, where each voxel had independently sampled noise.

The effective SNR vs size of ROI in voxels can be seen in figure 9. It ranged from around 140 for the smallest ROI, to around 9000 for the biggest ROI. Percentual errors and coefficients of variation for each metric are shown in figures 10 and 11.

To further assess the robustness of the ROI based fit, a second simulation was performed. While in the first ROI simulation every voxel shared the same ground truth, in the second simulation, each voxel had an independent “ground truth” curve, to which noise was then added (variable ground truth). The decay parameters for each curve were randomly selected from the interval [0.5**GT*, 1.5**GT*], where *GT* are the tumour ground truth parameters (obtained from the ROI fit of the *in vivo* data). The relative error plots to each parameter’s ground truth are shown in figure 12.

**Figures**


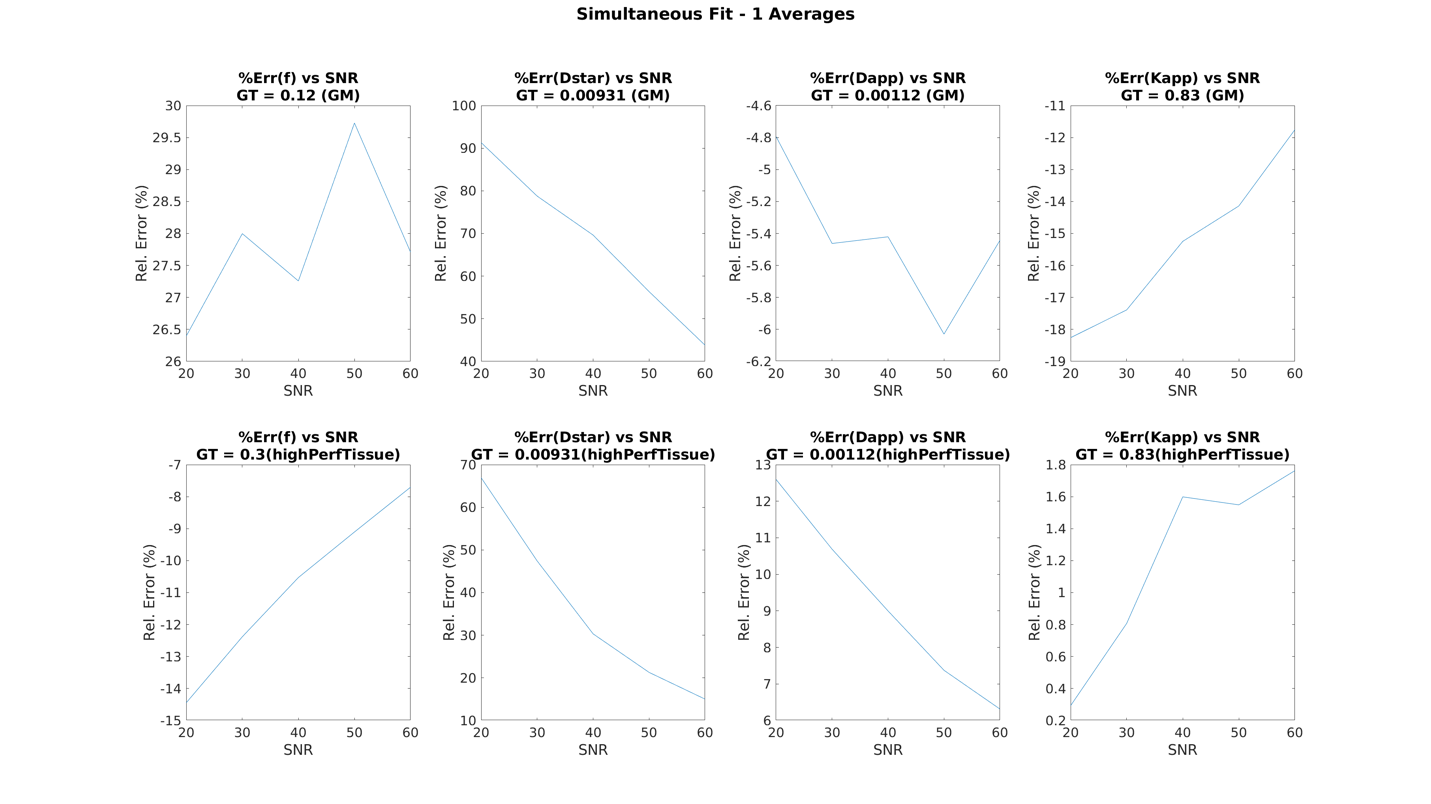


Figure 1 - Relative error of fitting parameters for clinical SNR range in GM.


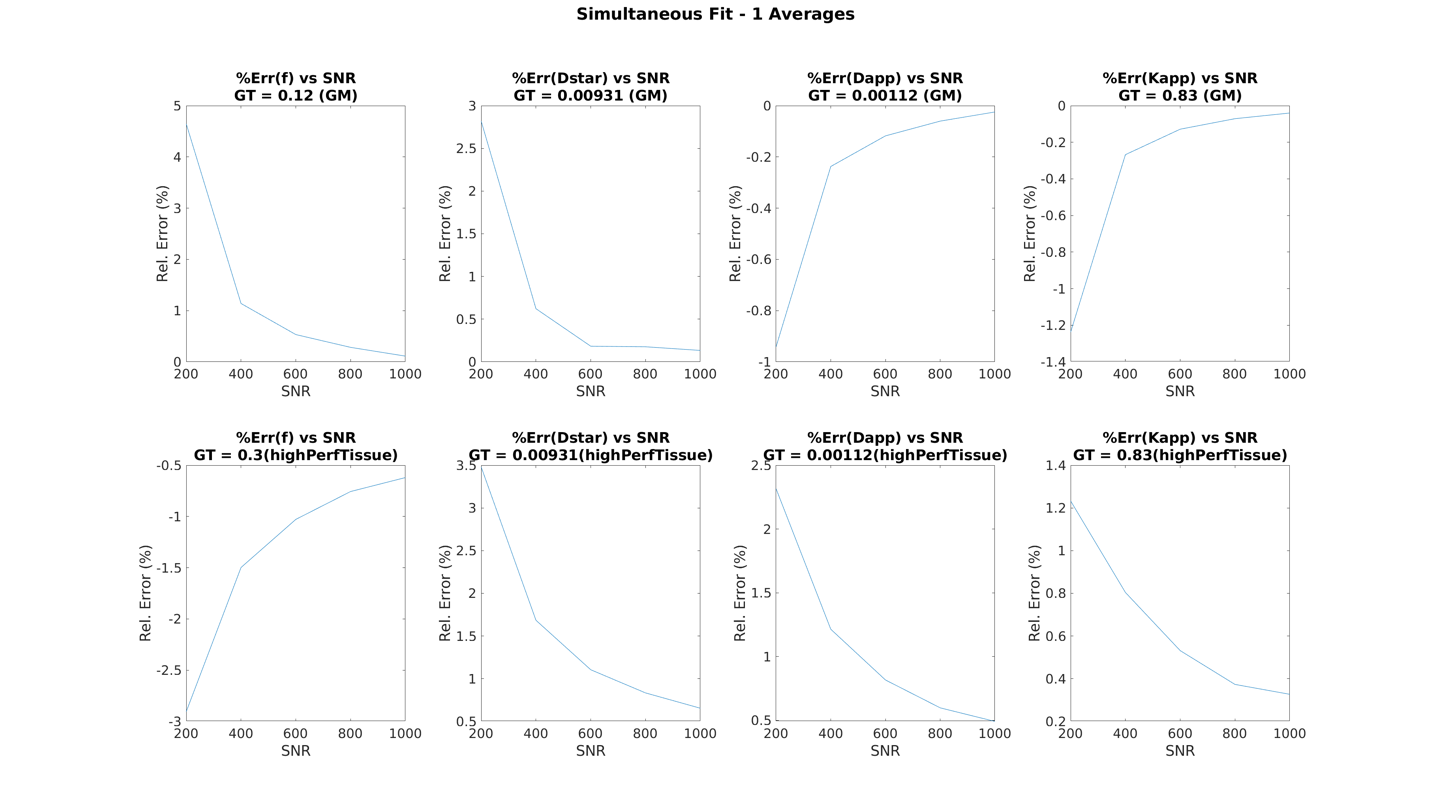


Figure 2 – Relative error of fitting parameters for high SNR range in GM.


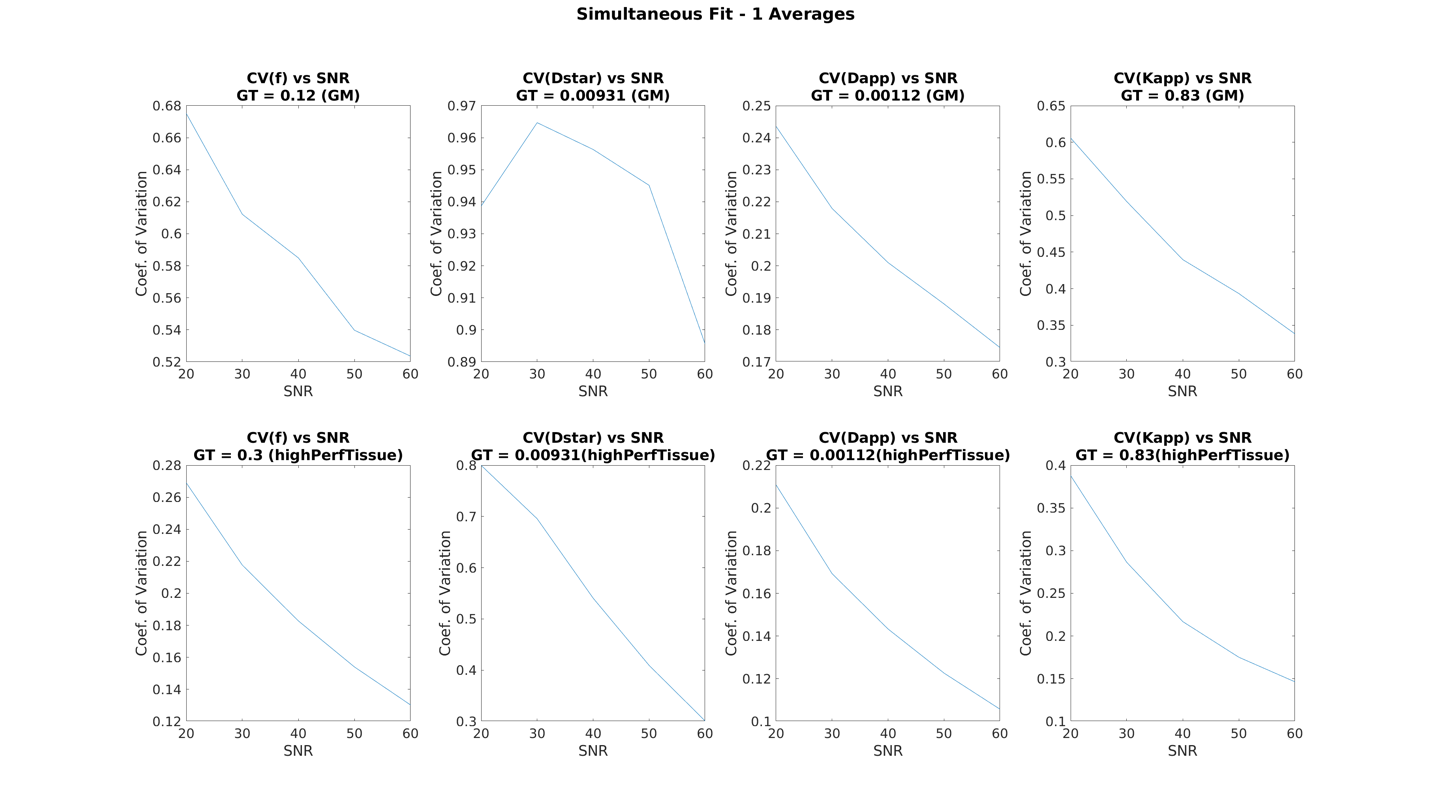


Figure 3 – Coefficient of variation of fitting parameters for the clinical SNR range in GM.


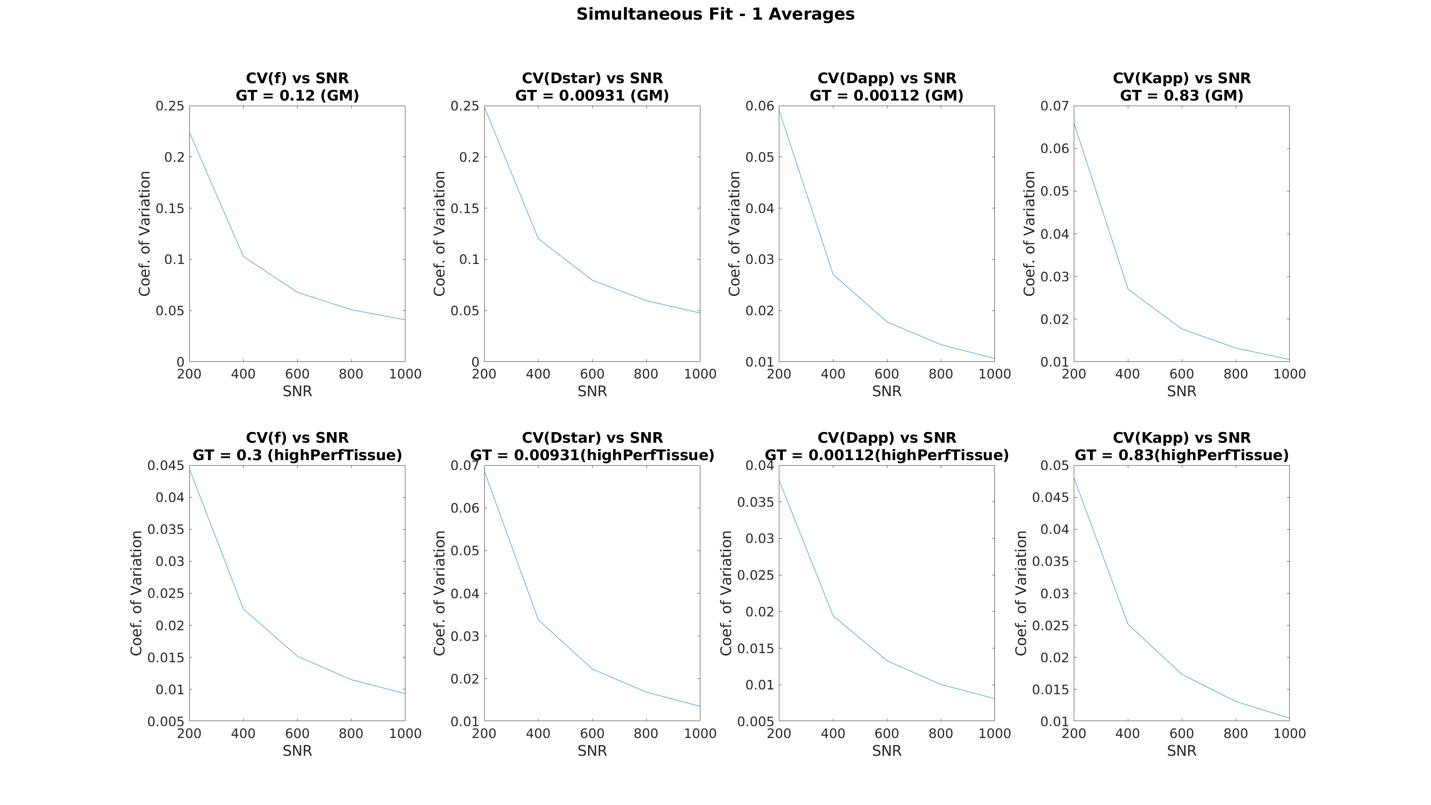


Figure 4 – Coefficient of variation of fitting parameters for the high SNR range in GM.


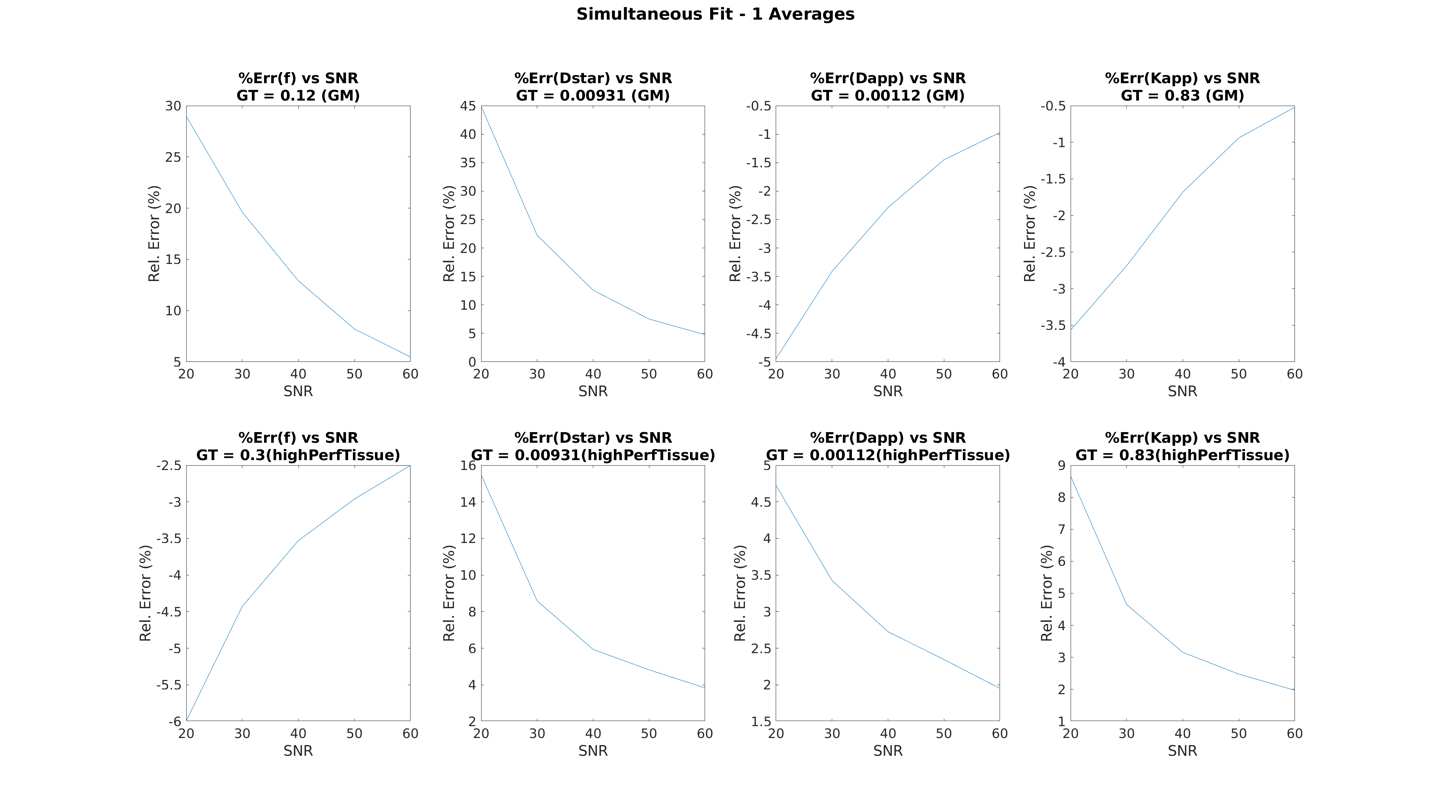


Figure 5 – Relative error of fitting parameters using the dense b-value scheme at clinical SNR range in GM.


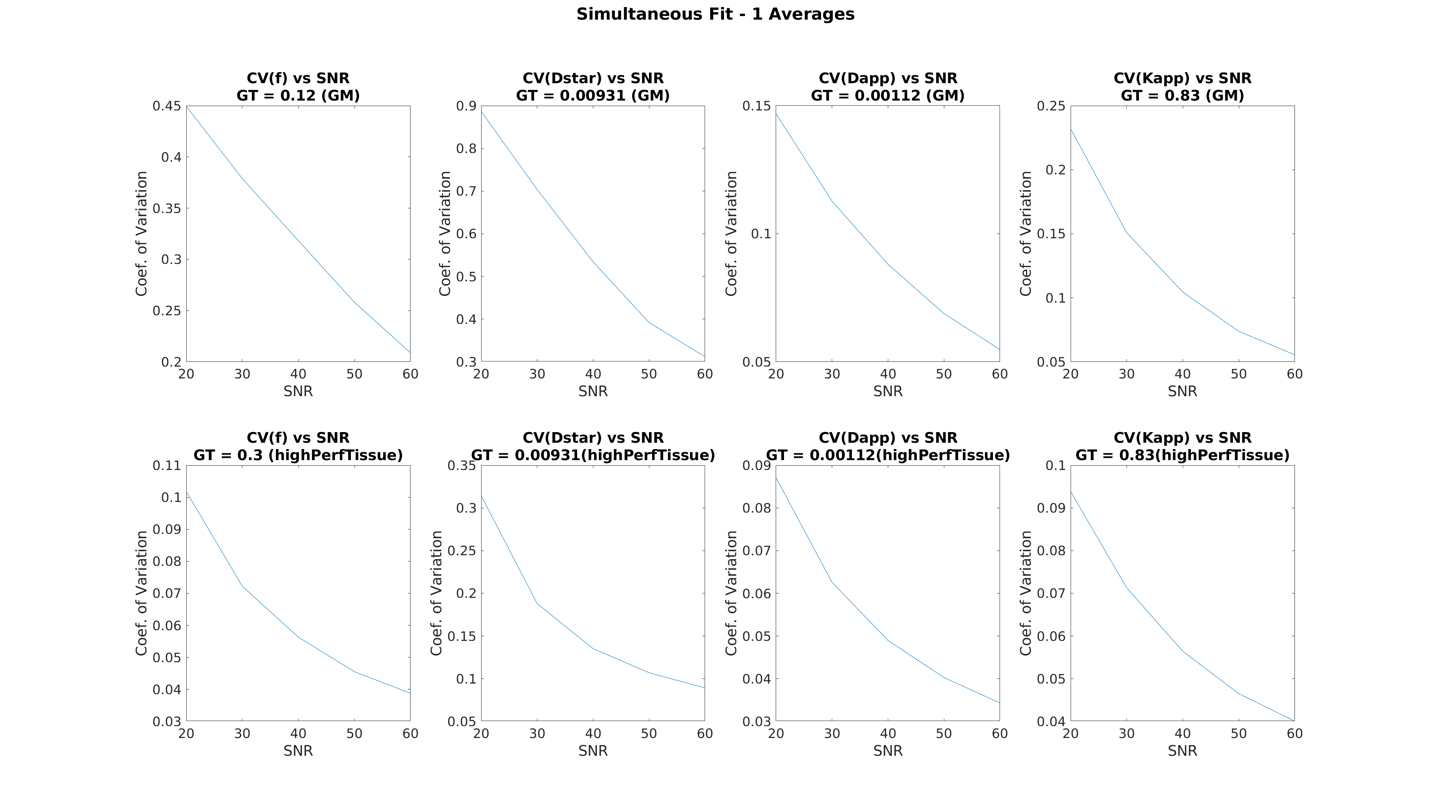


Figure 6 – Coefficient of variation of fitting parameters using the dense b-value scheme at clinical SNR range in GM.


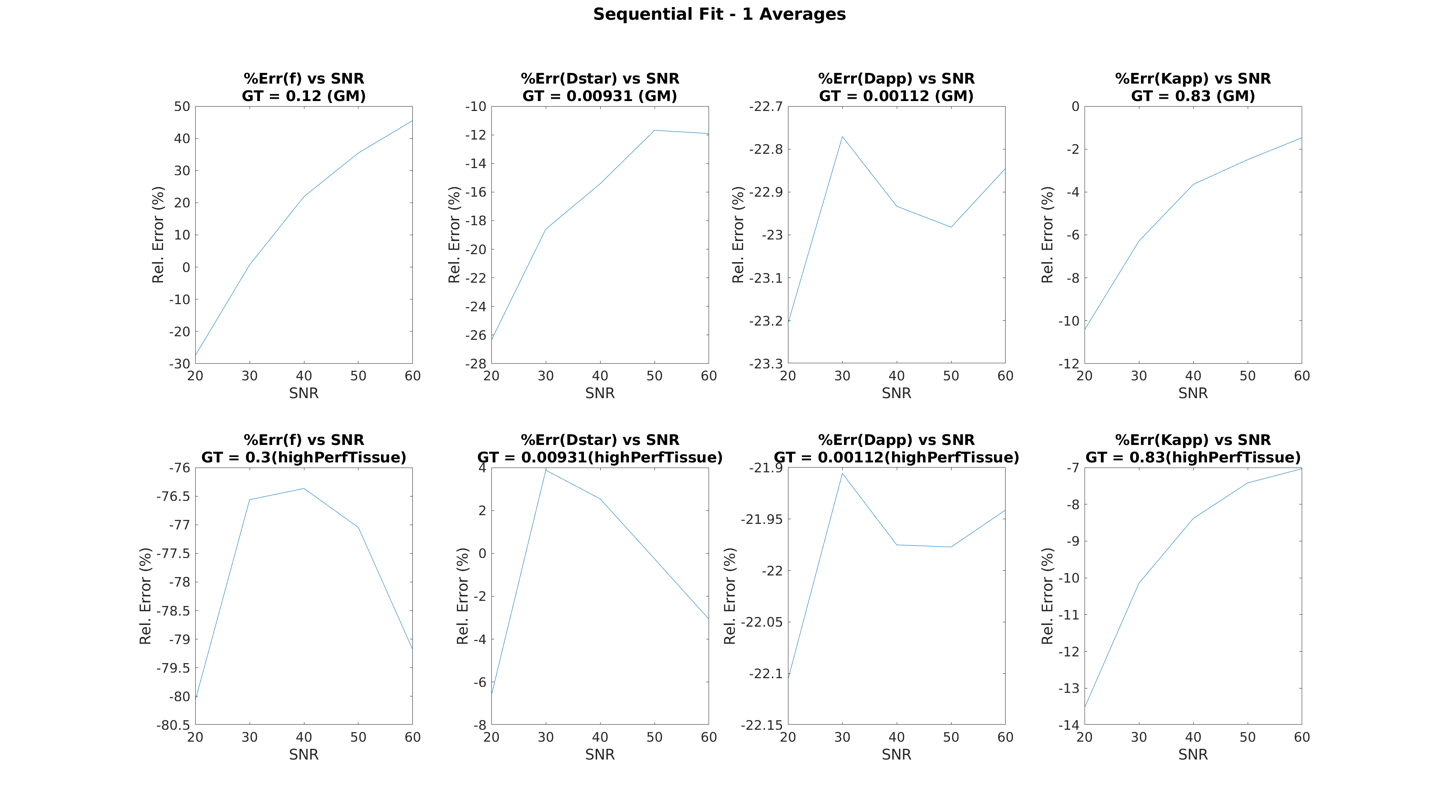


Figure 7 – Relative error of fitting parameters in GM (top row) and fictitious high perfusion tissue (bottom row), at clinical SNR and with the proposed sampling scheme.


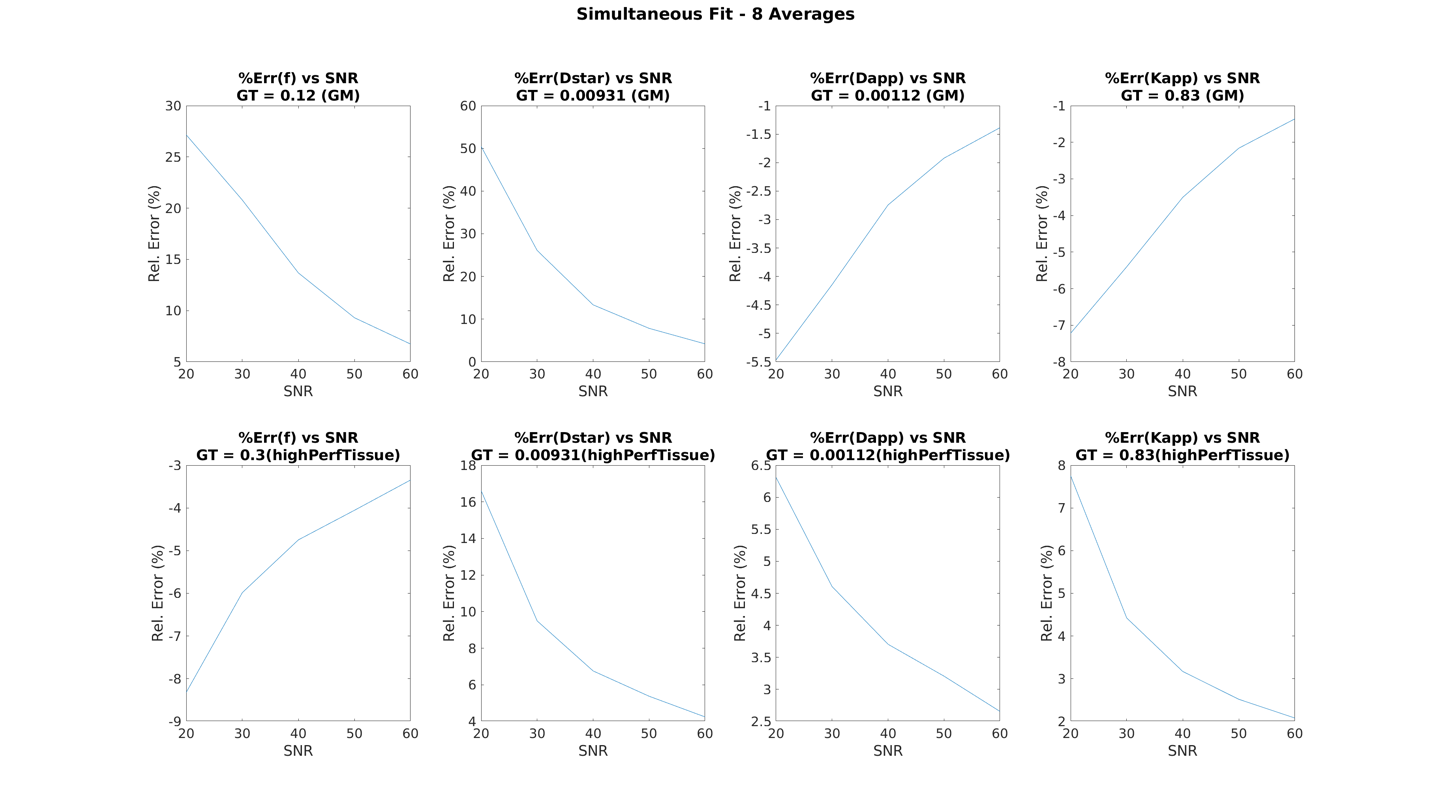


Figure 8 – Relative error in fitting parameters using 8 averages, clinical SNR range and the proposed b-value scheme.


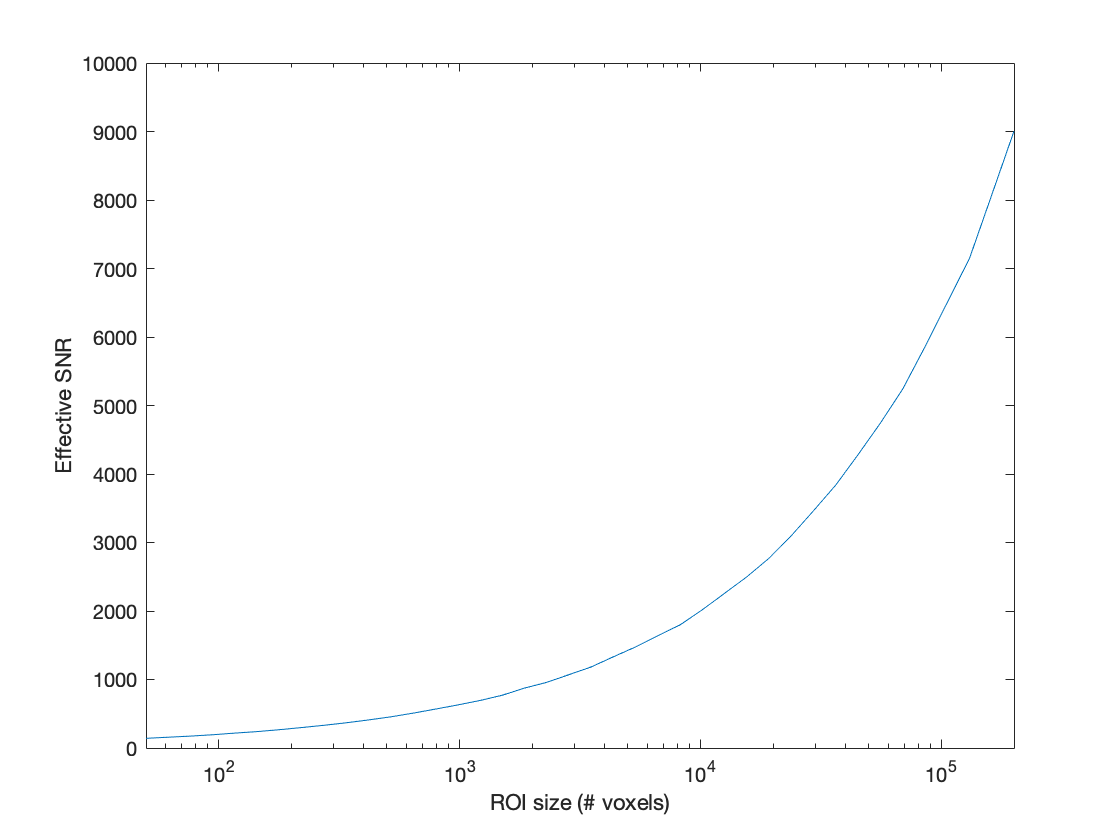


Figure 9 – Semi-logarithmic plot of the effective SNR vs ROI size in voxels.


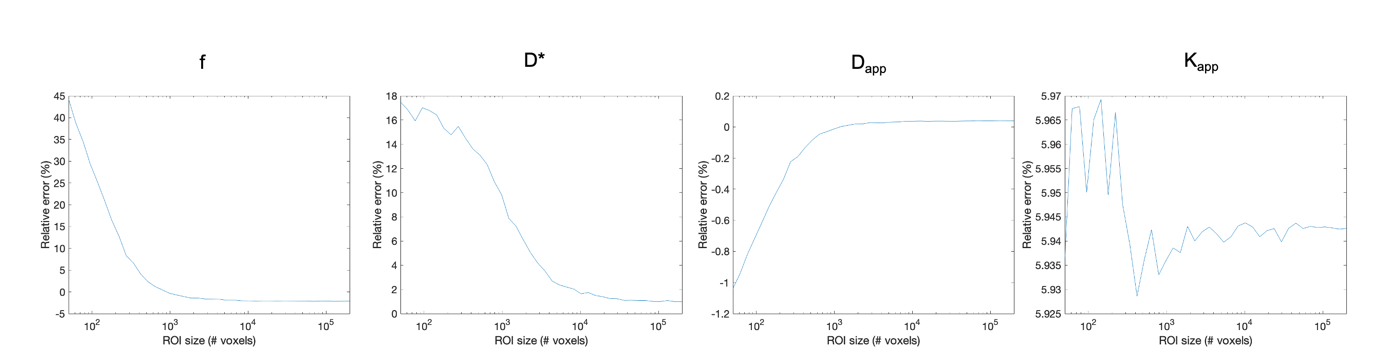


Figure 10 – Semi-logarithmic plot of the relative error vs ROI size in voxels


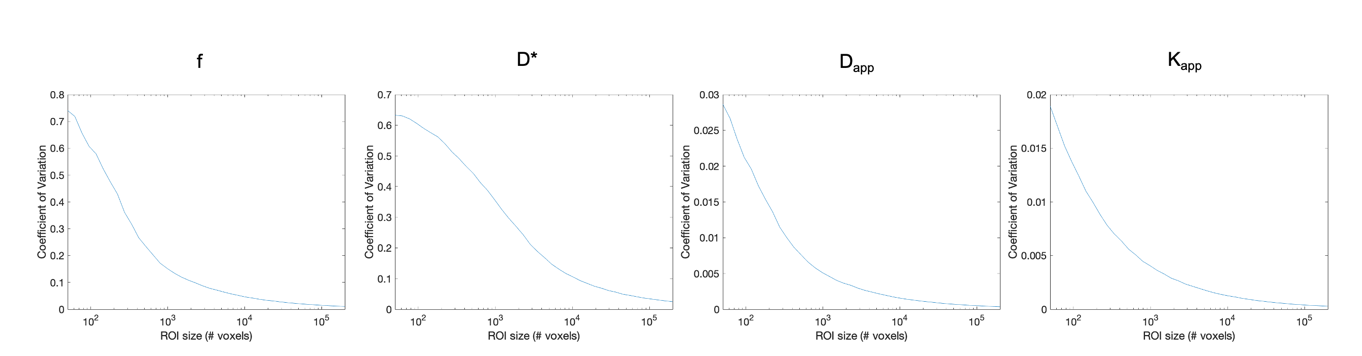


Figure 11 – Semi-logarithmic plot of the coefficient of variation vs ROI size in voxels


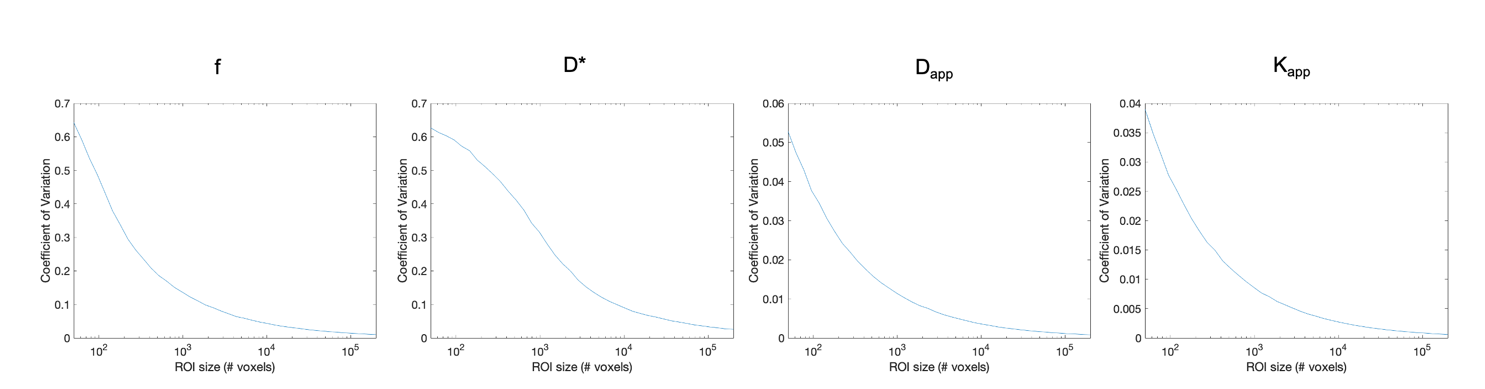


Figure 12 – Semi-logarithmic plot of the coefficient of variation vs ROI size for the random ground truth simulations
