# Supplementary material for: A Fast Protocol for Multiparametric Characterisation of Diffusion in the Brain and Brain Tumours
Source: Front Oncol. 2021 Sep 21;11:554205. doi: 10.3389/fonc.2021.554205 (PMC8490752; doi:10.3389/fonc.2021.554205)
Supplement: Supplementary file 3 [file Presentation_1.pptx]

## Slide 1
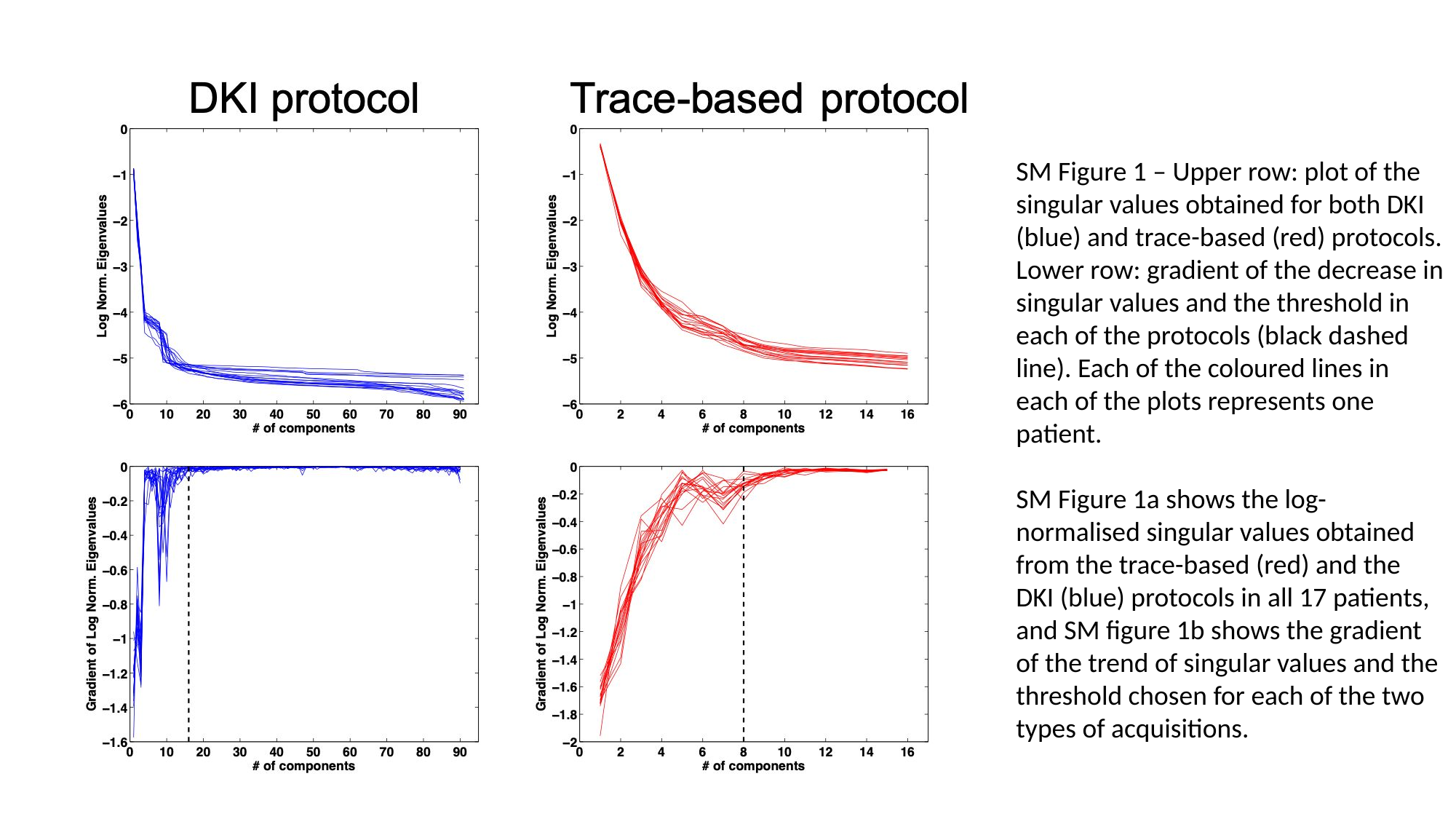

SM Figure 1 – Upper row: plot of the singular values obtained for both DKI (blue) and trace-based (red) protocols. Lower row: gradient of the decrease in singular values and the threshold in each of the protocols (black dashed line). Each of the coloured lines in each of the plots represents one patient.
SM Figure 1a shows the log-normalised singular values obtained from the trace-based (red) and the DKI (blue) protocols in all 17 patients, and SM figure 1b shows the gradient of the trend of singular values and the threshold chosen for each of the two types of acquisitions.

## Slide 2
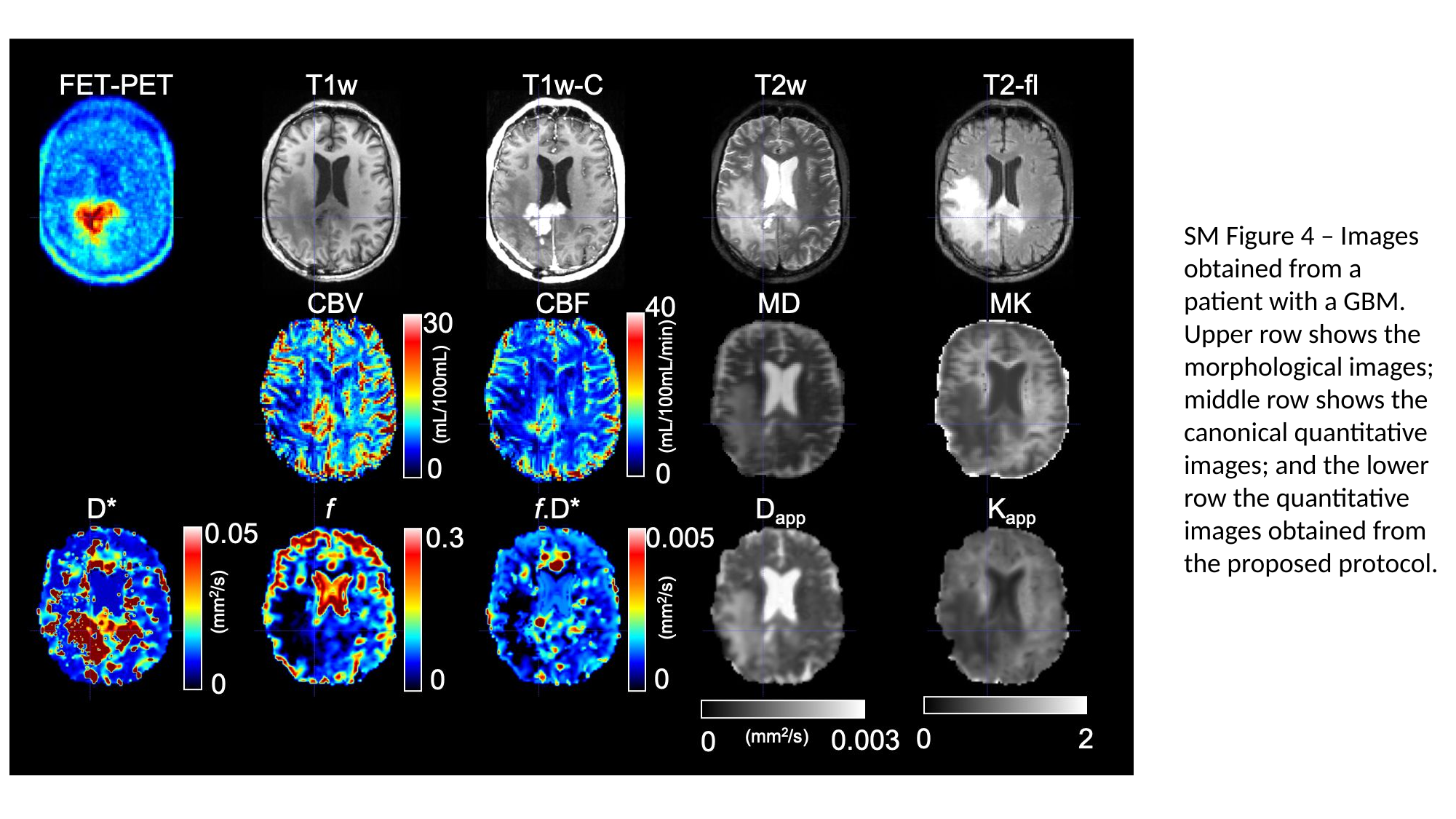

SM Figure 4 – Images obtained from a patient with a GBM. Upper row shows the morphological images; middle row shows the canonical quantitative images; and the lower row the quantitative images obtained from the proposed protocol.

## Slide 3
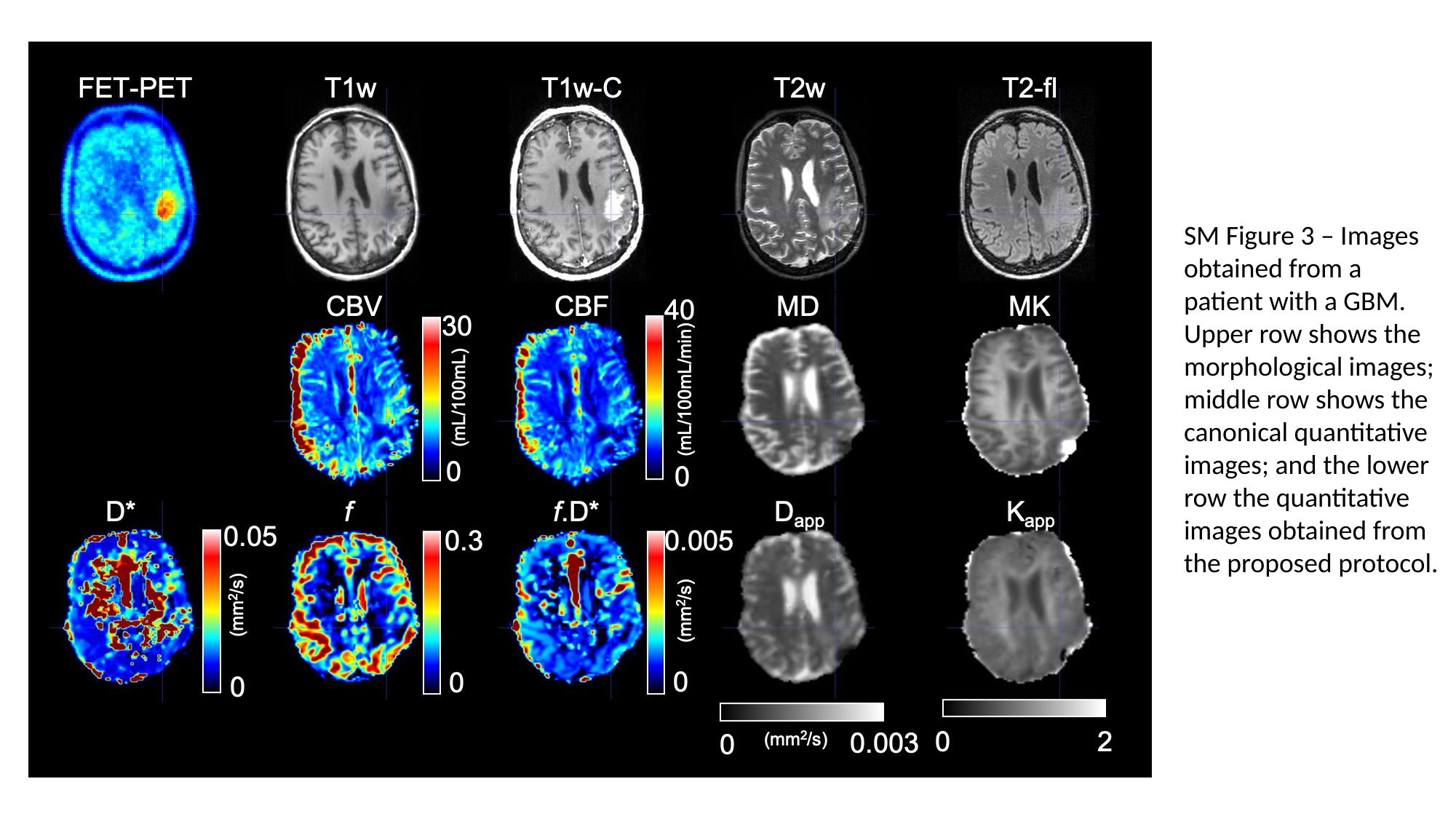

SM Figure 3 – Images obtained from a patient with a GBM. Upper row shows the morphological images; middle row shows the canonical quantitative images; and the lower row the quantitative images obtained from the proposed protocol.

## Slide 4
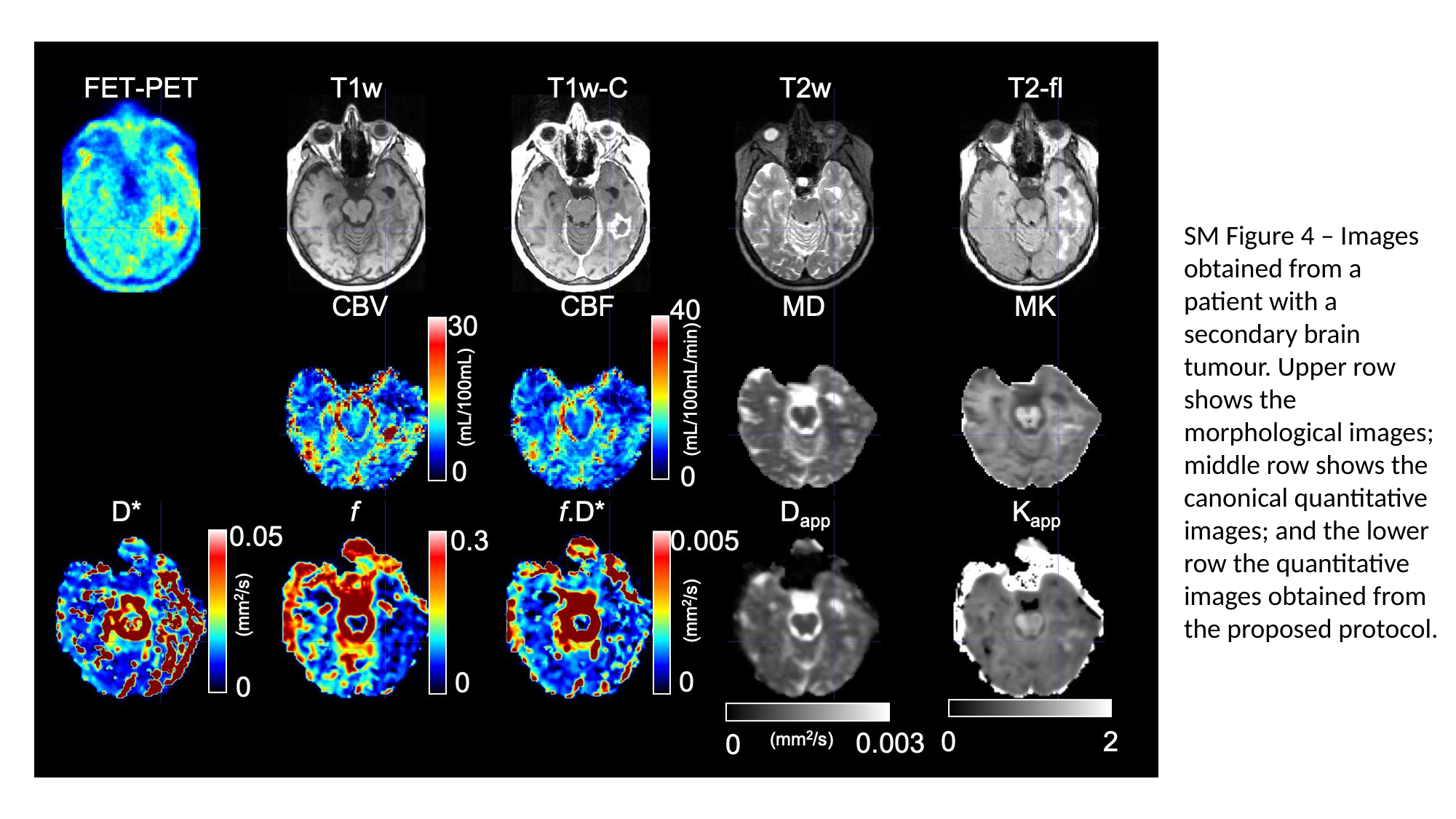

SM Figure 4 – Images obtained from a patient with a secondary brain tumour. Upper row shows the morphological images; middle row shows the canonical quantitative images; and the lower row the quantitative images obtained from the proposed protocol.

## Slide 5
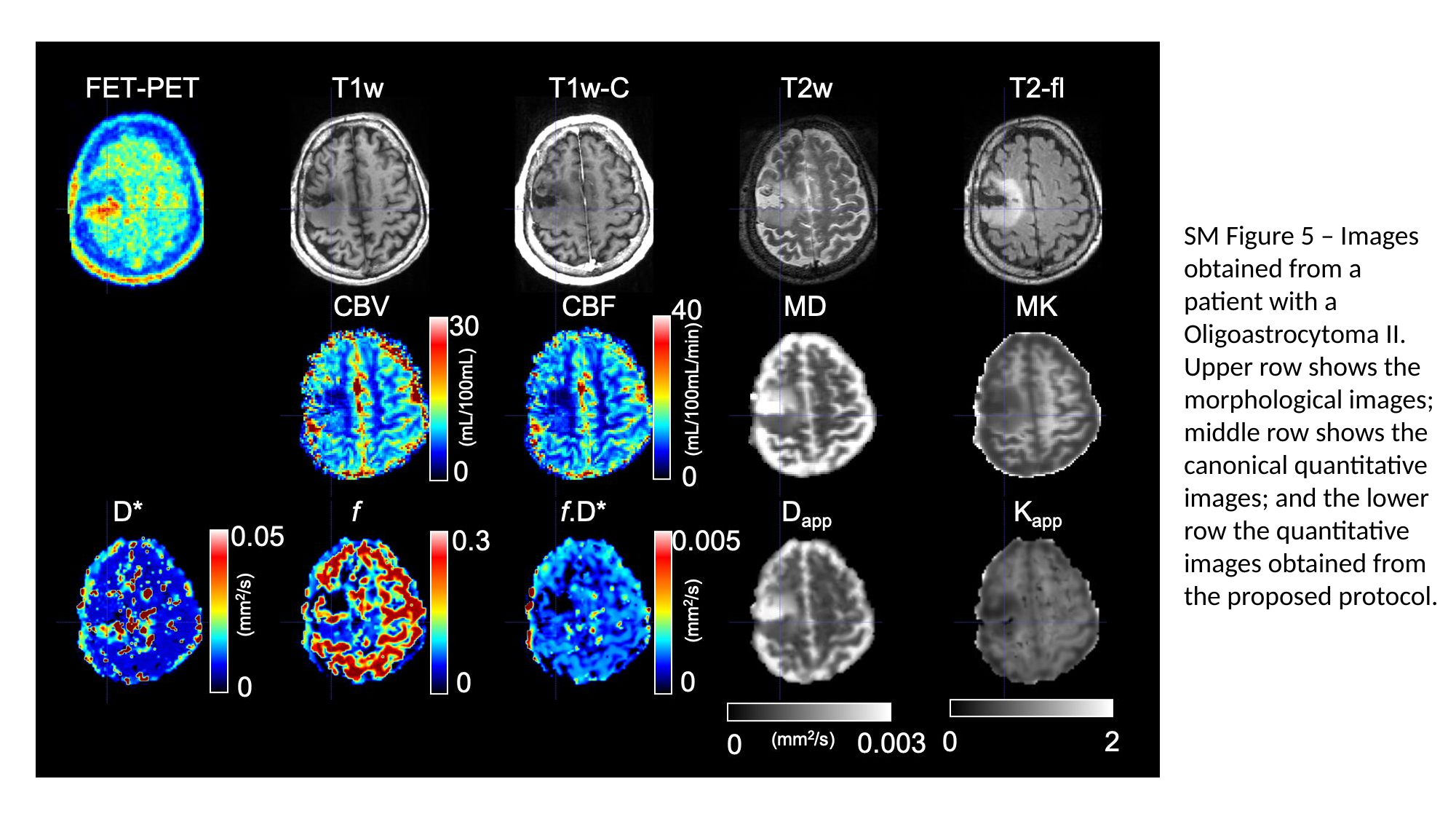

SM Figure 5 – Images obtained from a patient with a Oligoastrocytoma II. Upper row shows the morphological images; middle row shows the canonical quantitative images; and the lower row the quantitative images obtained from the proposed protocol.

## Slide 6
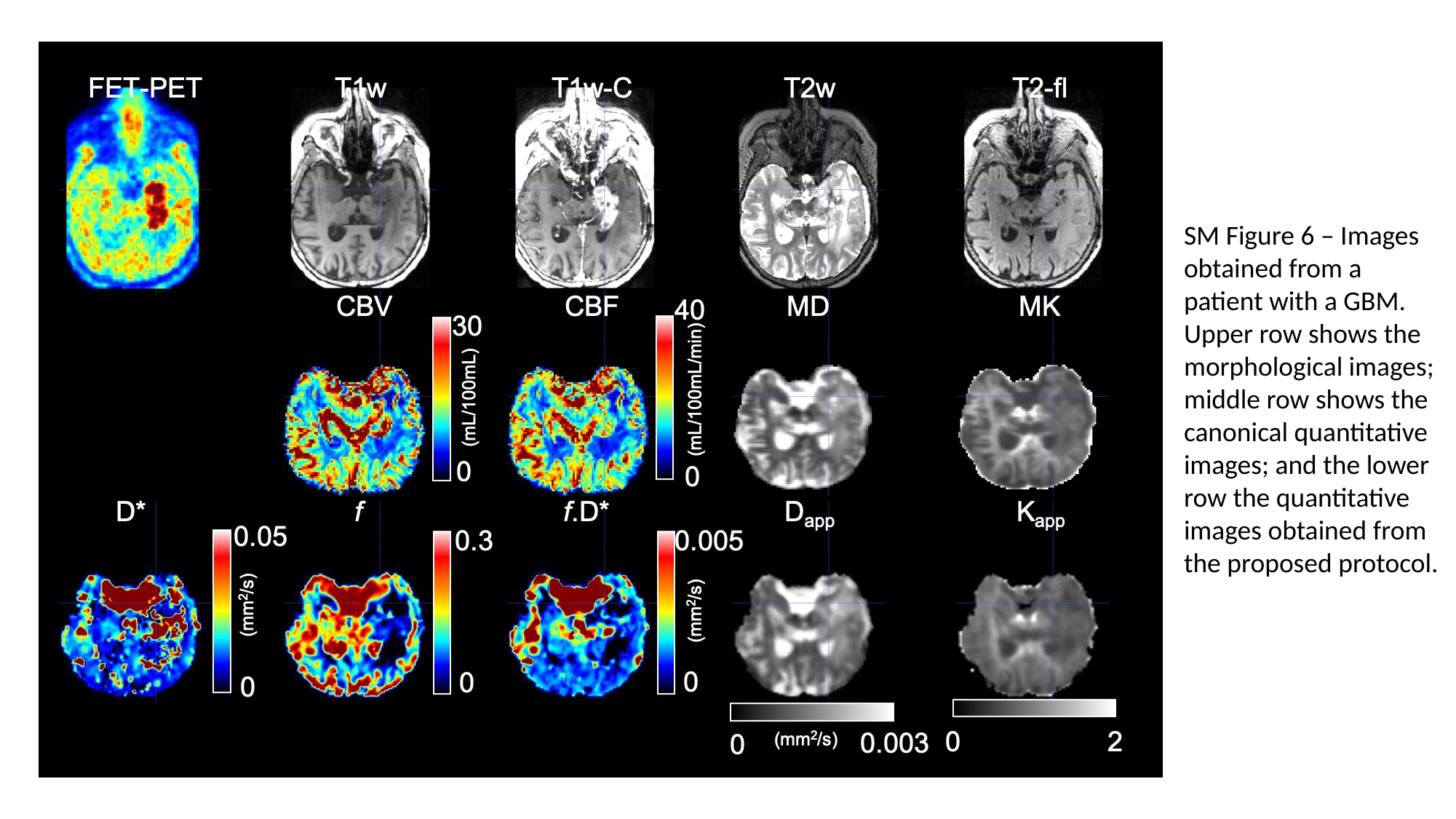

SM Figure 6 – Images obtained from a patient with a GBM. Upper row shows the morphological images; middle row shows the canonical quantitative images; and the lower row the quantitative images obtained from the proposed protocol.

## Slide 7
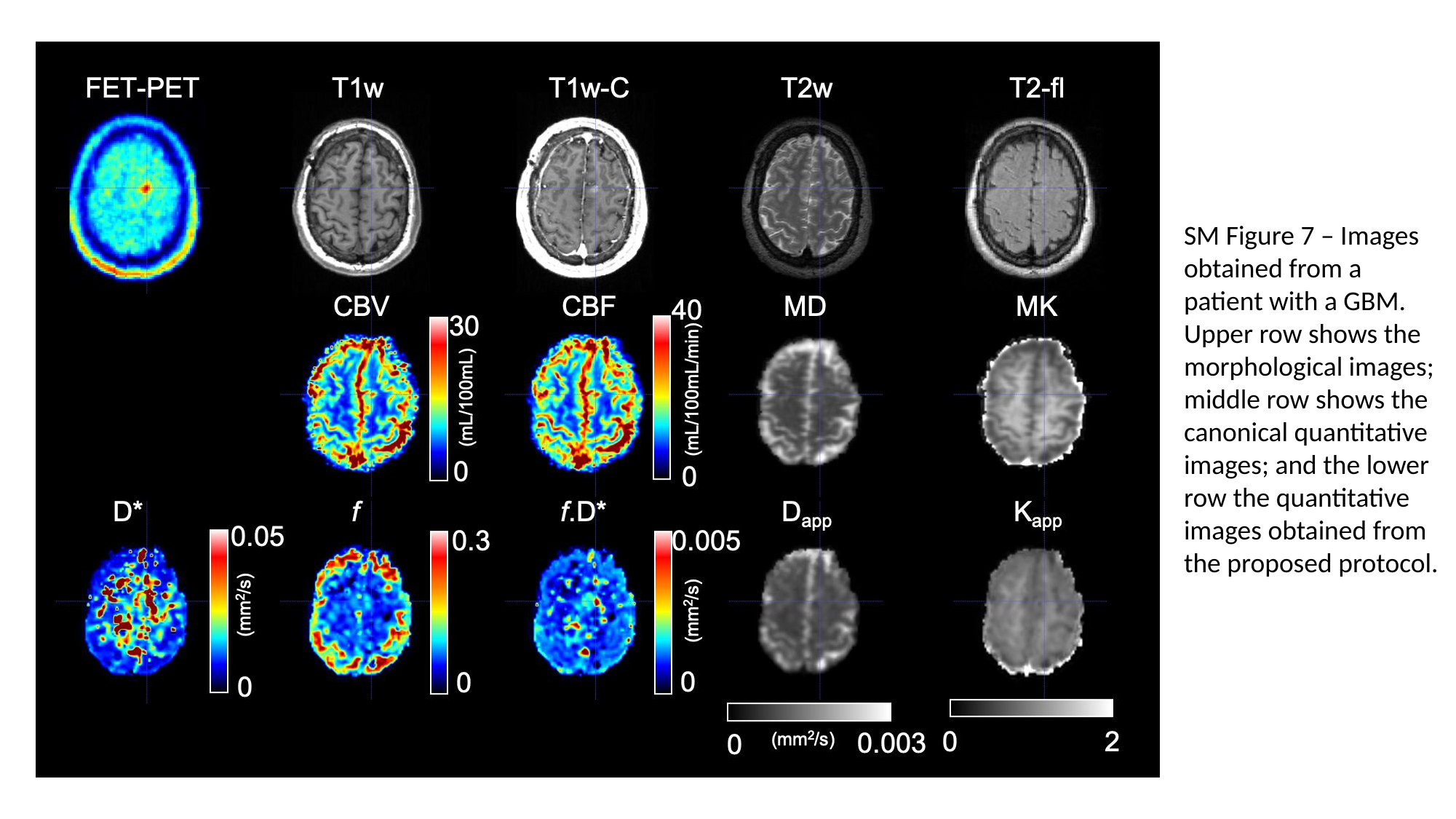

SM Figure 7 – Images obtained from a patient with a GBM. Upper row shows the morphological images; middle row shows the canonical quantitative images; and the lower row the quantitative images obtained from the proposed protocol.

## Slide 8
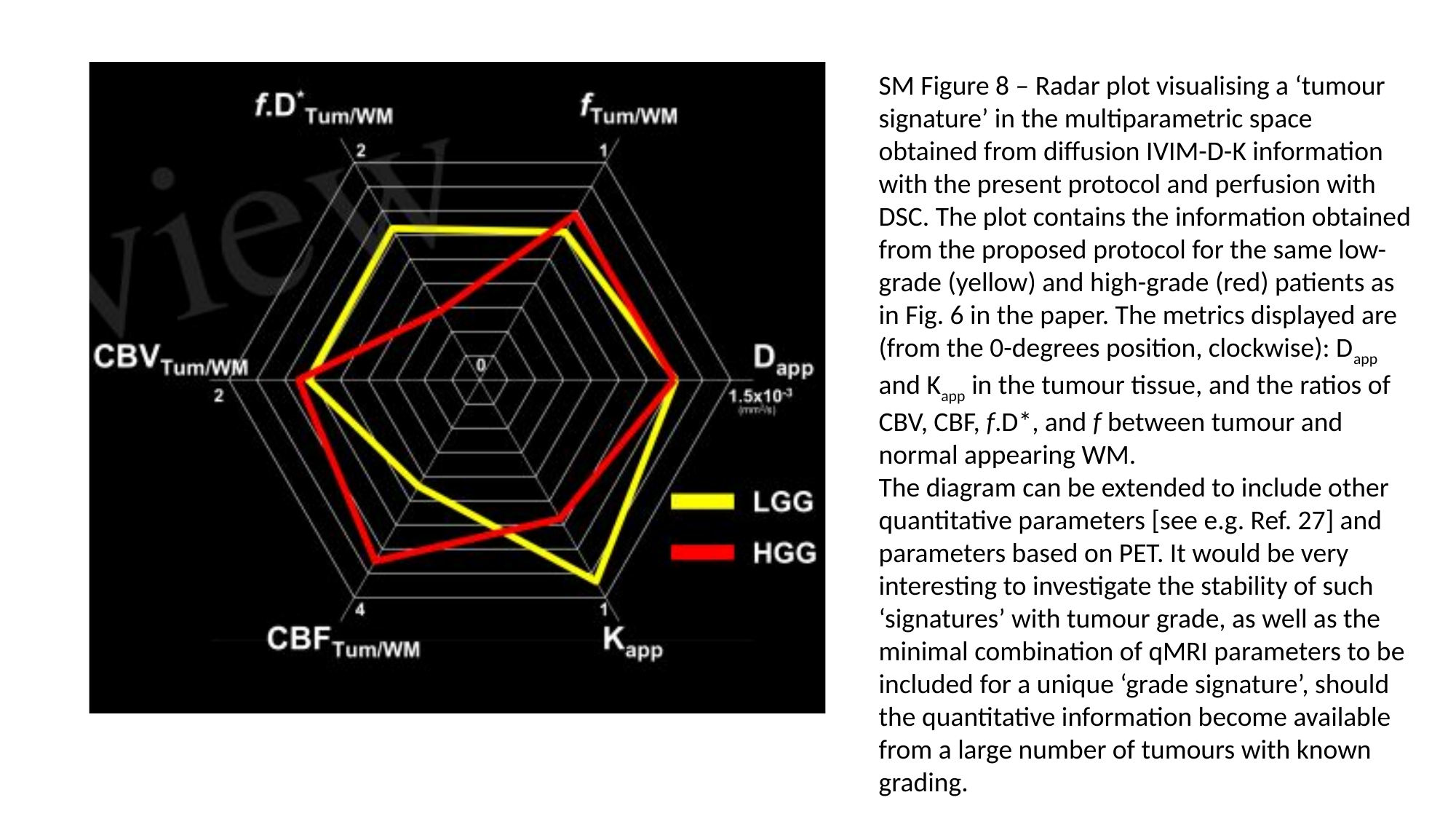

SM Figure 8 – Radar plot visualising a ‘tumour signature’ in the multiparametric space obtained from diffusion IVIM-D-K information with the present protocol and perfusion with DSC. The plot contains the information obtained from the proposed protocol for the same low-grade (yellow) and high-grade (red) patients as in Fig. 6 in the paper. The metrics displayed are (from the 0-degrees position, clockwise): Dapp and Kapp in the tumour tissue, and the ratios of CBV, CBF, f.D*, and f between tumour and normal appearing WM.
The diagram can be extended to include other quantitative parameters [see e.g. Ref. 27] and parameters based on PET. It would be very interesting to investigate the stability of such ‘signatures’ with tumour grade, as well as the minimal combination of qMRI parameters to be included for a unique ‘grade signature’, should the quantitative information become available from a large number of tumours with known grading.
